# Guideline for schizophrenia: implementation status and attitude toward an upcoming living guideline

**DOI:** 10.1007/s00406-023-01568-z

**Published:** 2023-02-18

**Authors:** Naiiri Khorikian-Ghazari, Carolin Lorenz, Duygu Güler, Theresa Halms, Astrid Röh, Marisa Flick, Angelika Burschinski, Charline Pielenz, Eva Salveridou-Hof, Thomas Schneider-Axmann, Marco Schneider, Elias Wagner, Peter Falkai, Wolfgang Gaebel, Stefan Leucht, Alkomiet Hasan, Gabriele Gaigl

**Affiliations:** 1 Department of Psychiatry, Psychotherapy and Psychosomatic, Medical Faculty, University of Augsburg, BKH Augsburg, Augsburg, Germany; 2 Department of Psychiatry and Psychotherapy, School of Medicine, Technical University of Munich, Medical Faculty, Klinikum Rechts der Isar, 81675 Munich, Germany; 3 Department of Psychiatry and Psychotherapy, LVR-Klinikum Düsseldorf, Medical Faculty, Heinrich-Heine-University, Düsseldorf, Germany; 4 WHO Collaborating Centre on Quality Assurance and Empowerment in Mental Health, DEU-131 Düsseldorf, Germany; 5 Department of Psychiatry and Psychotherapy, University Hospital, LMU Munich, Munich, Germany

**Keywords:** Living guideline, Guideline implementation, Schizophrenia

## Abstract

The implementation status of clinical guidelines is, despite their important role in connecting research with practice, frequently not satisfactory. This study aims to investigate the implementation status of the current German guideline for schizophrenia. Moreover, the attitude toward a living guideline has been explored for the first time by presenting screenshots of the German schizophrenia guideline transferred to a digital living guideline format called MAGICapp. A cross-sectional online survey was performed under the participation of 17 hospitals for psychiatry and psychosomatic medicine in Southern Germany and one professional association for German neurologists and psychiatrists. 439 participants supplied sufficient data for analysis. 309 provided complete data sets. Regarding the current guideline for schizophrenia and key recommendations, a large awareness-to-adherence gap was found. Group comparisons between different professions (caregivers, medical doctors, psychologists/psychotherapists, psychosocial therapists) detected differences in the implementation status showing higher awareness and agreement with the schizophrenia guideline and its key recommendations among medical doctors compared to psychosocial therapists and caregivers. Moreover, we detected differences in the implementation status of the guideline as a whole and its key recommendations between specialist and assistant doctors. The attitude toward an upcoming living guideline was mostly positive, especially among younger healthcare professionals. Our findings confirm an awareness-to-adherence gap, not only for the current schizophrenia guideline in general but also for its key recommendations with apparent differences between professions. Overall, our results show promising positive attitudes toward the living guideline for schizophrenia among healthcare providers, suggesting that a living guideline may be a supportive tool in everyday clinical practice.

**Supplementary Information:**

The online version contains supplementary material available at 10.1007/s00406-023-01568-z.

## Background

The implementation of practical guidelines in routine clinical practice is a challenge. It is a well-known problem that in spite of a multitude of existing clinical guidelines, the implementation in clinical practice remains insufficient worldwide [1–4]. For this reason, the focus of research has increasingly shifted from the development to the implementation of guidelines [5].

However, why is the implementation of clinical guidelines so important? Non-maleficence is one of the four basic principles in medical ethics. It is impossible in clinical practice to never do harm; however, decision-making in healthcare requires a thorough consideration of doing no net harm [6]. Evidence-based guidelines are intended to support clinicians in making decisions, not only by ensuring that harm is prevented, but also by providing optimal healthcare [6]. However, studies in different countries show that many patients receive care that is not needed or even potentially harmful [3, 7, 8]. Moreover, guidelines support optimizing health outcomes [5, 9–13]. This illustrates the importance of improving the implementation of guidelines in clinical practice.

In 2019, the German evidence- and consensus-based S3 guideline for schizophrenia [14, 15] was published. A recent preceding study conducted in the year of publication of the guideline showed that in spite of a high acceptance of this guideline, more than half of the participants did not use the guideline in everyday clinical care [16].

One reason for these results are the multifaceted barriers and facilitating factors in guideline adherence [5, 16–18]. Several frameworks exist allocating the barriers and facilitators to different categories. One of them is the in 1996 by Pathman and colleagues developed awareness-to-adherence model [19], a four-step model including awareness, agreement, adoption, and adherence. It postulates that a sequence of cognitive and behavioral processes is necessary so that guidelines can have an impact on physicians’ clinical behavior. Clinicians follow practical guidelines if they are aware of the guidelines, intellectually agree with them and then decide to follow them for some patients. Finally, the path to guideline adherence also requires regular adherence to it for most patients. A progressive drop off over the four steps is observed in general, which is described as a ‘pipeline’, with research evidence ‘leaks’ leading to a reduced guideline implementation [20].

A further reason is the fact that medical knowledge is increasing exponentially; consequently, many guidelines are already out of date when published [21–25]. Moreover, most clinicians cannot keep up with the amount of increasing knowledge [21, 26]. One strategy to address this problem is the development of so-called living guidelines. Living guidelines are an optimization of the guideline development process as individual recommendations can be updated as soon as relevant new evidence is available [22]. In that regard, the user’s perspective on the concept of living guidelines has not yet been investigated.

This study aims to explore the current implementation status of the German evidence-and consensus-based guideline for schizophrenia [14, 15] and its key recommendations as well as the attitude of users toward a future living guideline for schizophrenia*.*

## Design and methods

### Subjects and recruitment

**Fig. 1:**
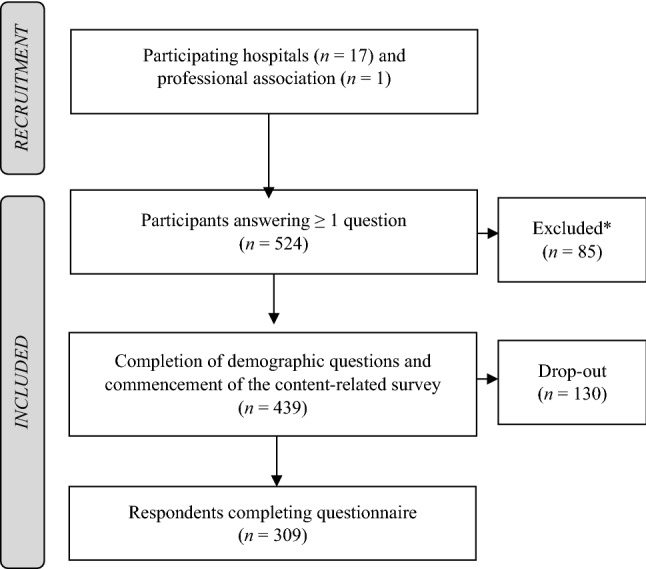
Recruitment and study flow chart. *Participants were excluded due to missing experience on the treatment of mental disorders (*n* = 22) or the absence of answering at least one content-related question (*n* = 63)

The cross-sectional online survey was performed from 01/2022 to 04/2022. In total, 17 hospitals for psychiatry, psychotherapy, and psychosomatic medicine in Southern Germany (see Supplementary Table 1) and one professional association for German neurologists and psychiatrists (BVDN: Berufsverband Deutscher Nervenärzte e. V.) participated in the study by forwarding the link to the survey to their clinical staff/members [27]. We recruited caregivers of the participating hospitals in the same manner as the other professions. After approximately three weeks, a reminder mail was sent. The licensed LimeSurveyR version 5.3.4+ (LMU hospital) was used to generate the questionnaire, conduct the survey, and to ensure an anonymous participation. Figure 1 displays the recruitment and study flow chart. The data protection officer of the University Hospital Munich reviewed the survey and the local ethical committee approved the project (reference number 21-0780). The trial has been performed according to the latest version of the Declaration of Helsinki [28]. If not stated otherwise, the term “schizophrenia guideline” refers to the current German evidence-and consensus-based guideline for schizophrenia (as of 2019) [14].

### Survey structure

The survey aimed to identify the compliance (a) of the general guideline for schizophrenia and (b) of four-key recommendations (see Supplementary Table 2) using the concept of an adapted awareness-to-adherence questionnaire by Pathman et al. [19]. The four recommendations are listed in Supplementary Table 2 and were selected in advance by the editors of the German schizophrenia guideline based on their high-evidence and recommendation levels and practical importance (dose of antipsychotics, antipsychotics for relapse prevention, management of severe weight gain, and application of cognitive behavioral therapy).

Regarding the guideline as a whole and the four key recommendations, participants were asked if they were familiar, agreed with the guideline/recommendation, assessed it as appropriate and feasible and how high they estimated the percentage of patients receiving treatment according to the guideline/respective recommendation. In our analysis, we assigned Pathman’s four categories to these four questions, i.e., awareness, agreement, adoption, and adherence.

Respondents were classified as aware if they were “familiar” or “very familiar” (five-point Likert scale) with the guideline (question 13) or a specific recommendation (questions 17, 21, 25, 32), respectively. Agreement was assessed by participants indicating whether they agreed with the guideline (question 14)/recommendation (questions 18, 22, 26, 30), nominal scale: “yes” versus “no”. They were classified as (potential) adopters if they regarded the guideline (question 15)/recommendation (questions 19, 23, 27, 31) as appropriate and feasible (five-point Likert scale: “agree” and “fully agree”). Finally, those who indicated that 90% or greater of their patients received a treatment according to the guideline (question 16)/the specific recommendation (questions 20, 24, 28, 32) were considered as adherers. Additionally, the attitude toward the living guideline for schizophrenia was investigated on a five-point Likert scale (agreement: 1 = strongly disagree, 3 = neutral, 5 = strongly agree) by assessing user-friendliness (questions 33, 34, 36), the perceived practicability (questions 37, 38, 39) as well as preferences in the application and potential benefits as compared to the print version (questions 40, 41). At the time of the study, no living guideline for mental disorders was available [29]. Therefore, we introduced the concept of a living guideline by an explanatory text and presented screenshots of the digitally prepared guideline for schizophrenia within the MAGICapp system, which is an evidence ecosystem making the whole process of a living guideline possible [30].

With regard to the implementation status of the guideline for schizophrenia, we investigated contrasts between the different professions: medical doctors, psychologists/psychotherapists, psychosocial therapists, and caregivers. For medical doctors, we additionally explored contrasts between specialist doctors and assistant doctors. Concerning the attitude toward the living guideline, we had a closer look at age differences assuming difference in digital competence across age groups [31]. Please see the supplement for the provided text and examples as well as the whole questionnaire in the supplement. The questionnaire was provided in German language and translated in English by the authors for this publication (see supplement).

### Statistical analysis

All analyses were carried out in IBM SPSS for Windows (version 28) with a significance level of *α* = 0.05. Descriptive statistics are displayed with frequency and percentage distributions for binary data. For continuous data, means and standard deviations are presented and additionally medians for categorical data. Intergroup differences were assessed using Chi^2^ tests in case of binary data. For categorical data (e.g., Likert scale), Mann–Whitney-*U* tests, Kruskal–Wallis tests for between group analyses (Dunn–Bonferroni tests for subgroup analyses in case of significant intergroup differences) or Wilcoxon signed-rank tests (in case of dependent samples within subjects) were used. For continuous data, we applied independent sample *t*-tests or one-way ANOVAs (Bonferroni tests for subgroup analyses in case of significant intergroup differences, Welch tests homogeneity of variance was violated). In addition to age groups [young (20–34 years old) vs. middle-aged (35–49 years old) vs. older mental healthcare professionals (50–66 years old)], professional groups were compared. Therefore, we conducted intergroup comparisons between medical doctors, psychotherapists/psychologists, psychosocial therapists as well as caregivers. See Table 1 for a detailed listing of the associated occupational profiles.

**Table 1:** Descriptive characteristics of participants

	Total *N* = 439	
	*n*	%
*Gender*		
Female	299	68.1%
Male	140	31.9%
Divers	0	0.0%
*Profession*		
Psychologist/psychotherapist		
Total	80	18.2%
Psychological psychotherapist	24	5.5%
Psychotherapy trainee	40	9.1%
Psychologist	16	3.6%
Medical doctor		
Total	187	42.6%
Specialist for psychiatry and psychotherapy	94	21.4%
Assistant doctor for psychiatry and psychotherapy	78	17.8%
Specialist for psychosomatic medicine and psychotherapy	2	0.5%
Assistant doctor for psychosomatic medicine and psychotherapy	2	0.5%
Specialist for neurology	6	1.4%
Assistant doctor for neurology	3	0.7%
Specialist for general medicine with additional qualification for psychosomatic care	1	0.2%
Assistant doctor for general medicine with additional qualification for psychosomatic care	0	0.0%
Specialist/assistant doctor of other medical fields	1	0.2%
Psychosocial therapist		
Total	67	15.3%
Occupational therapist	27	6.2%
Sport therapist	10	2.3%
Social pedagogue	17	3.9%
Social worker	1	0.2%
Art therapist	12	2.7%
Peer/Recovery attendant	0	0.0%
Sociotherapist	0	0.0%
Caregiver		
Total	96	21.9%
Specialist nurse for psychiatric care	28	6.4%
Qualified nurse	64	14.6%
Remedial nurse (Heilerziehungspfleger:in)	4	0.9%
Other profession	9	2.1%
*Workplace/Setting*^a^		
Inpatient setting		
University hospital	69	15.7%
Public hospital	320	72.9%
Non-profit hospital	28	6.4%
Private hospital	14	3.2%
Outpatient setting		
Practice with health insurance license	7	1.6%
Private practice	4	0.9%
Practice within the framework of psychotherapy training	10	2.3%
Research	10	2.3%
Other	4	0.9%
		M (SD)
*Age*		
Years	439	41.41 (11.62)
	Mdn	*M* (SD)
*Experience*^b^		
Mental disorders	4.00	3.88 (0.91)
Schizophrenic disorders	3.00	3.43 (0.95)

*N* number of participants, *M* means, *SD* standard deviations, *Mdn* medians

^a^Multiple answers were possible

^b^Participants were asked how they would rate their experience in treating people with mental disorders or schizophrenic disorders (1 = not at all experienced—5 = very experienced)

## Results

### Participants’ characteristics

309 respondents finalized the survey ultimately. Of the 524 participants who initially participated (response to at least one question) in the survey, 439 mental healthcare professionals provided sufficient data for analyses. 85 participants were excluded from analyses due to missing experience in the treatment of mental disorders (*n* = 22) or the absence of answering at least one content-related question (*n* = 63). Moreover, 130 respondents dropped out along the questionnaire, leaving 309 respondents who completed the survey, see Fig. 1. Demographic information is shown in Table 1. Comparisons on demographic information between excluded and included participants, professions, specialist and assistant doctors as well as age groups are shown in the Supplementary Results (Supplementary Tables 3–6).

### Awareness, agreement, adoption, and adherence: implementation status of the guideline for schizophrenia and its key recommendations

In total, less than half of the participants were aware of (40%), agreed with (43%), and adopted (41%) the guideline for schizophrenia. Less than one-tenth (7%) of the surveyed mental healthcare professionals reported to adhere to the schizophrenia guideline as a whole. Regarding the specific recommendations, awareness ranged between 38% for recommendation 3 (severe weight gain) and 81% for recommendation 2 (relapse prevention). Likewise, recommendation 3 (severe weight gain) exhibited the lowest rates on agreement (36%), adoption (33%), and adherence (5%), whereas recommendation 2 (relapse prevention) received the highest rates on agreement (88%), adoption (74%), and adherence (40%). Throughout the recommendations, a large discrepancy between awareness and adherence as well as between agreement and adherence was found. The greatest awareness-to-adherence gap for all four professions together was detected for recommendation 4 (psychotherapy)—68% of the participants fell off the track from awareness to adherence and 74% agreed on the recommendation but did not adhere to it. For complete test statistics and an overview of the evaluated recommendations, see Table 2 and Supplementary Table 2, respectively.

**Table 2:** Mean response comparisons between professions regarding the implementation status (awareness, agreement, adoption, and adherence) of the guideline for schizophrenia in general as well as of four selected recommendations

	Total		PSY		MED		PST		CG		Chi-square test		
	*N*	%Yes	*N*	%Yes	*N*	%Yes	*N*	%Yes	*N*	%Yes	*X*^2^	*df*	*p*
*Awareness*													
Guideline for schizophrenia [Q13]	439	40.1%	80	23.8%	187	57.2%	67	22.4%	96	33.3%	42.30	3	< 0.001
Recommendation 1 [Q17]	409	79.0%	75	65.3%	182	94.5%	59	49.2%	84	82.1%	68.70	3	< 0.001
Recommendation 2 [Q21]	390	81.0%	73	75.3%	179	94.4%	56	53.6%	73	79.5%	51.61	3	< 0.001
Recommendation 3 [Q25]	376	37.8%	72	12.5%	174	59.2%	52	15.4%	70	28.6%	66.95	3	< 0.001
Recommendation 4 [Q29]	370	75.7%	71	88.7%	173	85.5%	49	53.1%	69	58.0%	41.97	3	< 0.001
*Agreement*													
Guideline for schizophrenia [Q14]	438	42.5%	80	31.3%	187	64.7%	67	17.9%	95	26.3%	68.57	3	< 0.001
Recommendation 1 [Q18]	409	86.1%	75	88.0%	182	96.7%	59	64.4%	84	81.0%	44.56	3	< 0.001
Recommendation 2 [Q22]	390	87.7%	73	93.2%	179	96.1%	56	60.7%	73	86.3%	54.31	3	< 0.001
Recommendation 3 [Q26]	376	36.2%	72	19.4%	174	52.3%	52	17.3%	70	27.1%	38.83	3	< 0.001
Recommendation 4 [Q30]	370	81.1%	71	93.0%	173	87.3%	49	59.2%	69	72.5%	30.26	3	< 0.001
*Adoption*													
Guideline for schizophrenia [Q15]	436	40.8%	51	51.0%	166	66.3%	28	42.9%	56	46.4%	11.38	3	0.010
Recommendation 1 [Q19]	409	70.2%	75	69.3%	182	80.8%	59	49.2%	84	66.7%	22.98	3	< 0.001
Recommendation 2 [Q23]	390	73.6%	73	68.5%	179	88.3%	56	48.2%	73	65.8%	42.30	3	< 0.001
Recommendation 3 [Q27]	375	32.8%	72	23.6%	174	46.6%	52	11.5%	69	26.1%	29.53	3	< 0.001
Recommendation 4 [Q31]	369	56.6%	71	78.9%	172	51.2%	49	53.1%	69	50.7%	17.64	3	< 0.001
*Adherence*													
Guideline for schizophrenia [Q16]	287	7.3%	51	7.8%	155	8.4%	27	0.0%	50	6.0%	2.60	3	0.458
Recommendation 1 [Q20]	326	23.9%	54	25.9%	165	26.7%	36	16.7%	68	16.2%	4.08	3	0.025
Recommendation 2 [Q24]	304	39.5%	53	34.0%	162	48.1%	32	12.5%	54	31.5%	17.01	3	< 0.001
Recommendation 3 [Q28]	218	4.6%	29	3.4%	138	5.1%	13	7.7%	36	2.8%	0.71	3	0.871
Recommendation 4 [Q32]	284	7.4%	58	10.3%	152	4.6%	28	10.7%	43	7.0%	3.00	3	0.392

*N* number of participants,), *%Yes* represents the percentage of participants, who were aware of, agreed on, adopted, or adhered to the recommendation, *df* degrees of freedom, *X*^2^ Chi-square value. Numbers of questions are displayed in square brackets. The complete questionnaire is shown in the supplement. *Total* all participants included, *PSY* psychologists/psychotherapists, *MED* medical doctors, *PST* psychosocial therapists, *CG* caregivers. For subgroup analyses, see Supplementary Table 7

Recommendation 1: Antipsychotics should be offered as low as possible and as high as necessary (lowest possible dosage) within the international consensus recommended dosage range. Particularly in first-episode patients, a low dose should be chosen as they are more sensitive to side effects and respond better to a lower dose. Recommendation 2: People with schizophrenia (first-onset and multiple-onset) should be offered treatment with antipsychotics for relapse prevention after evaluating individual risk–benefit. Recommendation 3: In cases of severe weight gain and the need to continue current antipsychotic medication, after implementation of psychotherapeutic and psychosocial interventions, treatment for weight loss should be offered by trying Metformin (first choice) or Topiramate (second choice) and by taking into account the risks for additional drug treatment. Recommendation 4: People with schizophrenia should be offered cognitive behavioral therapy

#### Group comparisons—profession

**Fig. 2:**
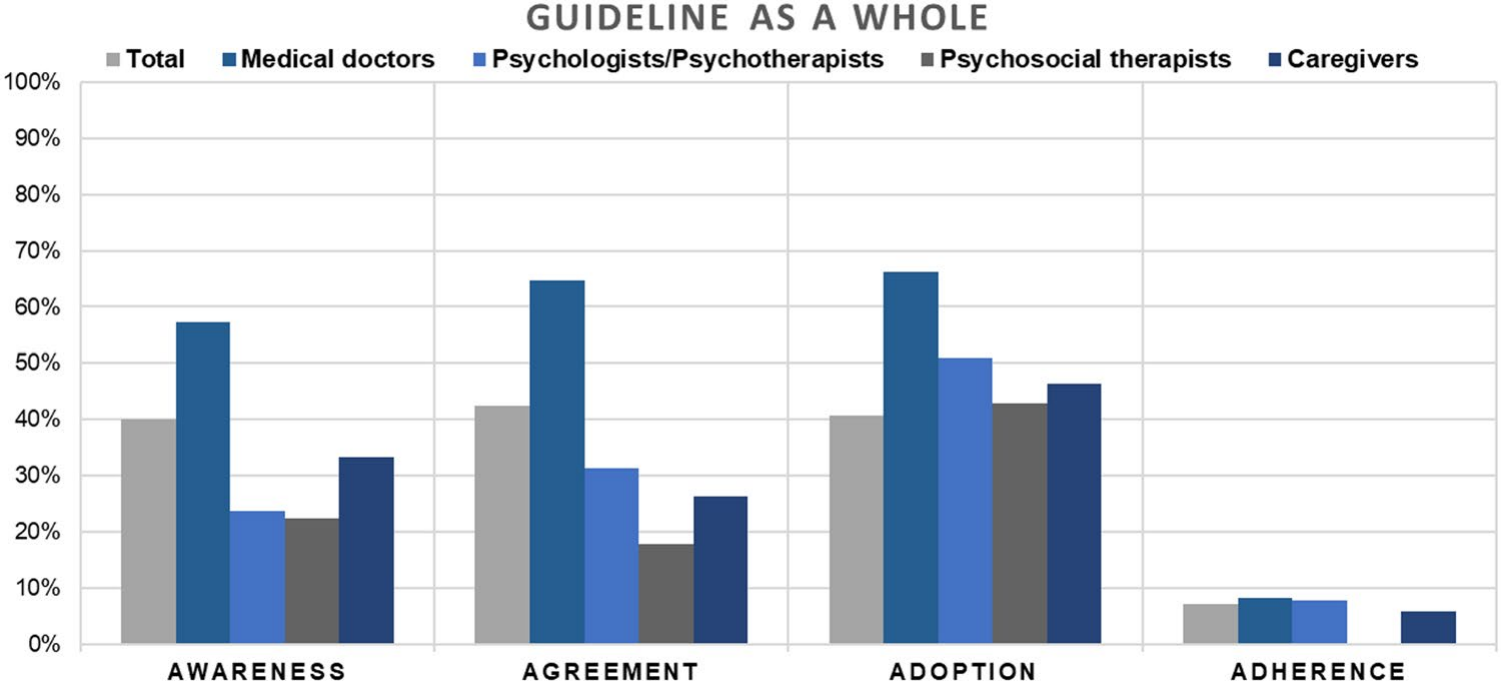
The bar chart shows the implementation status of the guideline as a whole among different professions (medical doctors, psychologists/psychotherapists, psychosocial therapists, caregivers). An awareness-to-adherence gap can be seen throughout all professions. Higher awareness and agreement rates of the current schizophrenia guideline as a whole can be detected among medical doctors compared to psychosocial therapists, psychologists/psychotherapists and caregivers (*ps* <0.001). For complete test statistics, see Table 2

**Fig. 3:**
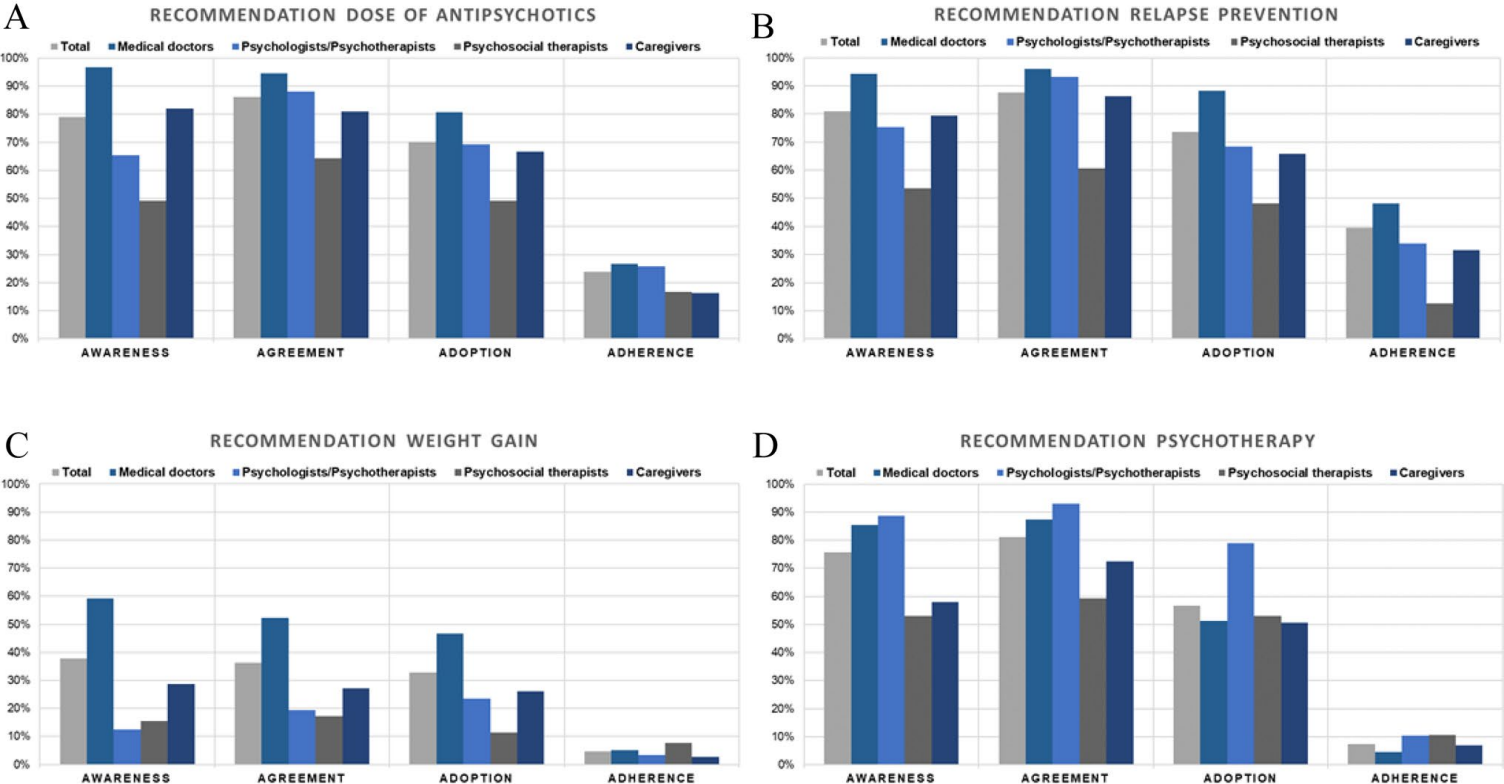
The bar chart depicts the implementation status of specific recommendations (**A**–**D**) among different professions (medical doctors, psychologists/psychotherapists, psychosocial therapists, caregivers). Throughout the recommendations, a large discrepancy between awareness and adherence as well as between agreement and adherence was found for all four professions (**A**–**D**). Higher awareness and agreement rates of all four recommendations can be detected among medical doctors compared to psychosocial therapists and caregivers (*ps* ≤ 0.036) (**A**–**D**). In A, the implementation status of the recommendation dose of antipsychotics and in B, the implementation status of the recommendation relapse prevention is shown. The recommendation weight gain (**C**) is the only one with significant differences among professions regarding adherence: medical doctors reported a higher adherence rate compared to psychosocial therapists, *p* = 0.001. In D, the implementation status of the recommendation psychotherapy is displayed. Psychologists/psychotherapists were more aware of and agreed more with the psychotherapy recommendation than psychosocial therapists and caregivers (*ps* ≤ 0.006). Moreover, psychologists/psychotherapists regarded the psychotherapy recommendation as more appropriate and feasible in the treatment of patients with schizophrenia (adoption) than any other profession (medical doctors, psychosocial therapists, caregivers), *ps* ≤ 0.018. For complete test statistics, see Table 2

Chi^2^ tests of independence indicate higher awareness and agreement rates of the current schizophrenia guideline as a whole as well as for all four recommendations among medical doctors compared to psychosocial therapists and caregivers (*p*s ≤ 0.036) (see Fig. 2). Additionally, psychologists/psychotherapists were more aware of and agreed more with recommendation 4 (psychotherapy) than psychosocial therapists and caregivers (*p*s ≤ 0.006). Moreover, psychologists/psychotherapists regarded recommendation 4 (psychotherapy) as more appropriate and feasible in the treatment of patients with schizophrenia (adoption) than any other profession (medical doctors, psychosocial therapists, caregivers), *p*s ≤ 0.018 (see Fig. 3). Regarding adherence, the estimated proportion of patients who receive a treatment according to the recommendation or the schizophrenia guideline as a whole, no significant differences between professions were found—except for recommendation 3 (relapse prevention): medical doctors reported a higher adherence rate compared to psychosocial therapists, *p* = 0.001. For complete test statistics, see Table 2.

#### Group comparisons—specialist versus assistant doctors

Chi^2^ tests of independence show higher awareness, agreement and adoption rates among specialist doctors for the schizophrenia guideline as a whole,* p*s ≤ 0.002 (see Supplementary Table 8). Moreover, specialist doctors stated to be more aware of recommendation 1 (dose of antipsychotics) and 4 (psychotherapy) and agreed more to recommendation 4 than assistant doctors, *p*s ≤ 0.022. Among assistant doctors, recommendation 3 (severe weight gain) exhibited higher agreement, adoption and adherence rates than among specialist doctors, *p*s ≤ 0.026.

### Attitude toward the living guideline for schizophrenia

To explore the attitude toward the living guideline for schizophrenia, three screenshots of the unpublished digital layout of the planned living guideline were presented for illustration. To give a broad overview, the table of contents, two examples of recommendations (social skills training and cognitive remediation) as well as a graphical comparison of two treatment options (e.g., to use for shared decision-making) were shown (Please see supplement for more details). About two-thirds (64%) of the surveyed mental healthcare professionals would currently, in the absence of an available living guideline, prefer the living guideline to the printed format, and three-quarters (75%) expressed awareness about the advantages of the living guideline over the printed version. Based on the presented images of the living guideline for schizophrenia, more than half of the participants regarded the living guideline as user-friendly—68% find the layout appealing and the content clearly presented, 77% can imagine getting along well with the living format and for 52% the living guideline seemed to be clearer than the previous print version. Regarding clinical practicability/relevance, more than half of the respondents considered the living guideline as more practical than the print format (62%) and as a valuable tool in everyday clinical practice (80%). However, the presented concept of a living guideline seemed to be more informative than the print version for only 39% of the surveyed mental healthcare professionals. See Table 3 for further descriptive information and the supplement for the description and presented pictures of the living guideline.

**Table 3:** Mean level of agreement: attitude toward the living guideline for schizophrenia

	*N*	%Yes	Mdn	*M* (SD)
*User-friendliness*				
I find the layout appealing and the content clearly presented. [Q33]	335	68.1	4.00	3.69 (0.83)
I can imagine getting along well with the living guideline. [Q34]	334	76.6	4.00	3.87 (0.76)
The living guideline seems clearer than the previous print version. [Q36]	335	51.9	4.00	3.61 (0.82)
*Clinical practicability/relevance*				
The living guideline seems to be more informative than the previous print version. [Q37]	334	39.2	3.00	3.40 (0.72)
The living guideline seems to be more practical than the previous print version. [Q38]	333	61.6	4.00	3.77 (0.80)
I can imagine that a living guideline would be a valuable tool in my everyday clinical practice. [Q39]	333	80.2	4.00	3.98 (0.76)
*General attitude*				
The advantages of a living guideline over a print version are evident to me. [Q40]	333	75.1	4.00	3.92 (0.82)
I would prefer a living guideline to the previous print version. [Q41]	334	63.8	4.00	3.80 (0.89)
Mean—positive attitude living guideline*				
	333	64.6	3.75	3.76 (0.57)

Agreement was assessed by a five-point Likert scale (1 = strongly disagree to 5 = strongly agree). *%Yes* represents the percentage of participants, who agreed or strongly agreed to the statement. *N* number of participants, *M* means, *SD* standard deviations, *Mdn* medians

Numbers of questions are displayed in square brackets. The complete questionnaire is shown in the supplement

*The variable represents the mean agreement level of the above displayed items

#### Group comparisons—age

Kruskal–Wallis tests showed significant differences between age groups concerning the attitude toward the living guideline. Young mental healthcare professionals (20–34 years old) showed a more positive attitude throughout all categories of a potential living guideline (user-friendliness, clinical practicability, general, mean value) than older participants (50–66 years old), *p*s ≤ 0.006, and a higher mean positive attitude than middle-aged respondents (35–49 years old), *p* = 0.003. No significant differences were found in the mean positive attitude between middle-aged and older participants, *p* = 0.291. For complete test statistics, see Table 4.

**Table 4:** Comparisons between age groups: attitude toward the living guideline for schizophrenia

	Young (20–34 years)			Middle-aged (35–49 years)			Older (50–66 years)			KWT		
	*N*	Mdn	M (SD)	*N*	Mdn	M (SD)	*N*	Mdn	M (SD)	*H*	*df*	*p*
*User-friendliness*												
I find the layout appealing and the content clearly presented. [Q33]	124	4.00	3.83 (0.86)	108	4.00	3.69 (0.84)	103	4.00	3.52 (0.74)	9.53	2	< 0.001
I can imagine getting along well with the living guideline. [Q34]	124	4.00	4.03 (0.78)	108	4.00	3.81 (0.76)	102	4.00	3.73 (0.68)	11.90	2	0.003
The living guideline seems clearer than the previous print version. [Q36]	124	4.00	3.81 (0.85)	108	3.00	3.54 (0.81)	103	3.00	3.43 (0.74)	10.20	2	0.006
*Clinical practicability/relevance*												
The living guideline seems to be more informative than the previous print version. [Q37]	124	4.00	3.61 (0.76)	108	3.00	3.33 (0.64)	103	3.00	3.23 (0.69)	14.17	2	0.001
The living guideline seems to be more practical than the previous print version. [Q38]	123	4.00	4.03 (0.87)	108	3.00	3.67 (0.70)	102	4.00	3.57 (0.75)	20.30	2	< 0.001
I can imagine that a living guideline would be a valuable tool in my everyday clinical practice. [Q39]	123	4.00	4.19 (0.80)	108	4.00	4.00 (0.76)	102	4.00	3.73 (0.63)	26.72	2	< 0.001
*General attitude*												
The advantages of a living guideline over a print version are evident to me. [Q40]	123	4.00	4.12 (0.83)	108	4.00	3.90 (0.84)	102	4.00	3.72 (0.75)	16.40	2	< 0.001
I would prefer a living guideline to the previous print version. [Q41]	123	4.00	4.04 (0.86)	108	4.00	3.80 (0.88)	103	4.00	3.52 (0.85)	18.77	2	< 0.001
Mean—positive attitude living guideline*												
	123	4.00	3.96 (0.60)	108	3.75	3.72 (0.54)	102	3.63	3.56 (0.51)	25.54	2	< 0.001

Agreement was assessed by a five-point Likert scale (1 = strongly disagree to 5 = strongly agree)

*N* number of participants, *M* means, *SD* standard deviations, *Mdn* medians, *KWT* Kruskal–Wallis test, *H* H value, *df* degrees of freedom. Number of question is displayed in square brackets

*The variable represents the mean agreement rate of the above displayed items. The complete questionnaire appears in the supplement. For subgroup analyses, see Supplementary Table 9

## Discussion

This study ascertained the current implementation of the German guideline for schizophrenia approximately three years after its publication in March 2019. It further provides an initial assessment of the attitude toward the German living guideline for schizophrenia (currently under development).

In a previous study, an insufficient implementation status of the guideline for schizophrenia was shown [16]. Beyond this, we investigated the implementation status of four key recommendations and differences in implementation between professions. As a living guideline for schizophrenia is currently under development, we examined the attitude toward a living guideline and discrepancies among different age groups.

Two-fifths of the participants were aware of, agreed with, and adopted [19] the guideline for schizophrenia, less than one-tenth of the surveyed mental healthcare professionals reported to adhere to the schizophrenia guideline as a whole, showing an awareness-, agreement-, as well as an adoption-to-adherence gap in guideline use. This result is consistent with findings of a study on the current German schizophrenia guideline conducted in 2019 directly after its publication [16], and other mental health or somatic guidelines [32, 33]. Similarly, a large discrepancy between awareness and adherence as well as between agreement and adherence was also shown for specific recommendations. The greatest awareness-to-adherence gap for all four professions together was detected for the psychotherapy recommendation—68% of the participants fell off the track from awareness to adherence and 74% agreed on the recommendation but did not adhere to it. A possible reason could be the lack of time [34] for psychotherapy in an inpatient setting due to work load and/or a lack of prioritization of psychotherapy compared to other treatment forms, e.g., pharmacological treatment. Moreover, recommendation 3 regarding severe weight gain exhibited the lowest rates on awareness, agreement, adoption, and adherence out of all recommendations in total. This may be the result of a lack of experience with prescribing the recommended metformin (Evidence level A) and concerns regarding side effects or interaction with other drugs. Another potential explanation is that the consequences of antipsychotic-induced weight gain are underestimated [35]. A close look regarding barriers and facilitators influencing the gap to adherence will, therefore, be of importance for further research.

Moreover, analyses showed significant differences between professions by showing higher awareness and agreement rates of the current schizophrenia guideline as a whole as well as for all four recommendations among medical doctors compared to psychosocial therapists and caregivers. Psychologists/psychotherapists were more aware and agreed more to the psychotherapy recommendation than psychosocial therapists and caregivers and regarded the recommendation as more appropriate and feasible in the treatment of patients with schizophrenia (adoption) than any other profession (medical doctors, psychosocial therapists, caregivers). Profession-specific differences in guideline implementation are in line with previously conducted studies in mental health as well as general health provision [16, 36] and highlight the need for greater attention to profession-specific barriers and facilitators in further research. The reasons for physicians’ higher awareness and agreement with the guideline may be that they usually represent the case-leading professional group in multiprofessional inpatient settings. Furthermore, these results could also be explained by the fact that the guideline as a whole contains a greater number of and more specific recommendations for medical doctors compared with other professional groups and that the majority of the experts involved in the development of the guideline are physicians [14].

The different curricula of the professions in Germany may play a substantial role. For example, medical doctors have a smaller proportion of psychotherapeutic content in their training compared to psychotherapists. Psychotherapists are taught various psychotherapeutic interventions during their studies, and this knowledge is expanded during three to five years of practical and theoretical training of psychotherapy. Medical doctors have far less modules on that topic, as psychotherapy is solely one component of a comprehensive specialist training program. It should be further investigated how the training/studies of various professions may differ regarding guidelines (e.g., guidelines are more used in training of medical doctors, and in training of psychotherapists, manuals are more predominant) and how this may influence the implementation of such.

Our results show higher awareness, agreement, and adoption rates among specialist doctors for the schizophrenia guideline as a whole compared to assistant doctors. Specialist doctors were not only more aware of the recommendation regarding dose of antipsychotics and psychotherapy but also agreed more to the latter recommendation than assistant doctors. This may be a reflection of the expertise associated with an increased professional experience.

On contrary, recommendation 3 (severe weight gain) exhibited higher agreement, adoption, and adherence rates among assistant doctors than specialist doctors showing further differences between the more experienced and usually older professionals and assistants. Further research is needed investigating whether a more recent education and/or the amount of experience influences the present results.

Our results indicate acceptance of the concept of a living guideline among the surveyed mental healthcare professionals—about two-thirds showed positive attitude toward the presented concept of a living guideline. More than half of the subjects evaluate the living guideline as clearer, more practical and generally preferable to the print version. However, less than half of the subjects regarded the living guideline as more informative than the print version. This implies an assumption among clinicians that the living format might not be an improvement regarding additional information content.

We detected significant differences between age groups on attitude toward the living guideline. Young mental healthcare professionals showed a more positive attitude throughout all categories than older participants did, and a higher mean positive attitude than middle-aged respondents did. An uncertainty dealing with new technology is very common in older professionals [31]. In addition, younger professionals tend to have a greater affinity for technology. Living guidelines are being increasingly used in practice [31] and could, therefore, be more incorporated into the training of younger practitioners. Further research should investigate if these findings are in agreement with perceived potential barriers and facilitating factors in the use of the upcoming living guideline for schizophrenia.

Limitations regarding the results of this study first include disadvantages related to online surveys, e.g., survey frauds, response bias, and lack of representativeness [37]. Due to data protection settings of the used software and the anonymous nature of our study, we did not implement tracking of IT addresses. Thus, we cannot exclude, e.g., that some participants participated more than one time. However, this scenario appears to be unlikely as our sample consisted of professionals from inpatient and outpatient settings of our model region and all participants were asked to answer the questionnaire only one time. Our setting differs significantly from, e.g., settings with recruitment from social media platforms where multiple participants are a relevant source of bias. However, our approach may have resulted in a sampling bias as specific clinics in the south of Germany were contacted to participate in the survey. Although the questionnaire is predominantly based on theoretical frameworks [19, 38], it was developed and adjusted for specific research questions, and thus be potentially biased by the researchers. Further, the reader should bear in mind that the living guideline is still under development and our participants received only a conceptual presentation. Moreover, significant differences in demographic information such as age, gender, work setting and experience with the treatment of schizophrenia between professions were detected. Additionally, significant differences between included and excluded participants could be identified concerning gender, profession (medical doctors and other professions), setting (public hospital, research, and other) as well as age (Supplementary Tables 3–6). Therefore, analyses should be interpreted with caution.

## Conclusion

Overall, our findings show a high number of non-adherers to the current guideline of schizophrenia. More specifically, a discrepancy between awareness and adherence, not only for the current schizophrenia guideline as a whole, but also for selected four key recommendations was found. Differences between professions were detected—medical doctors showed higher awareness and agreement on the guideline for schizophrenia as well as on its key recommendations compared to caregivers and psychosocial therapists. Therefore, the role of different profession-specific curricula should be considered in the efforts to increase guideline knowledge and acceptance and, consequently, in the process of implementation. In addition to profession-specific differences, our results indicate a crucial role of clinician’s experience in guideline implementation—higher rates of awareness, agreement, and adoption of the overall schizophrenia guideline were found among specialists compared to assistant doctors.

Overall, our results show promising positive attitudes toward the living guideline for schizophrenia among healthcare providers, suggesting that a living guideline may be a supportive tool in everyday clinical practice. Nevertheless, the development of new guideline formats (e.g., living) cannot address all challenges in guideline implementation. Yet, living guidelines can be helpful to provide more versatile, responsive, and, thus, more user-oriented guidelines (e.g., adapted to profession, experience level, age).

## Supplementary Information

Below is the link to the electronic supplementary material.

Supplementary file1 (DOCX 642 KB)

## Data Availability

The datasets used and/or analyzed during the current study are available from the corresponding author on reasonable request.
