# Supplementary material for: Guideline for schizophrenia: implementation status and attitude toward an upcoming living guideline
Source: Eur Arch Psychiatry Clin Neurosci. 2023 Feb 18;273(7):1587–98. doi: 10.1007/s00406-023-01568-z (PMC10465681; doi:10.1007/s00406-023-01568-z)
Supplement: Supplementary file 1 — Supplementary file1 (DOCX 642 KB) [file 406_2023_1568_MOESM1_ESM.docx]

***Supplement***

# **Supplementary Methods**

## ***1.1 Subjects and recruitment***

17 hospitals for psychiatry, psychotherapy and psychosomatic medicine in Southern Germany and one professional association for German neurologists and psychiatrists (BVDN: Berufsverband Deutscher Nervenärzte e. V.) participated in the study. In Supplementary Table 1 the corresponding hospitals are listed.

| Supplementary Table 1. List of participating hospitals and links to the respective homepages. |
| --- |

| 1.     Klinik für Psychiatrie und Psychotherapie, LMU | <https://www.lmu-klinikum.de/psychiatrie-und-psychotherapie> |
| --- | --- |
| 2.     Klinik für Psychiatrie und Psychotherapie, TU München | <https://www.mri.tum.de/psychiatrie-und-psychotherapie> |
| 3.     BKH Augsburg, Klinik für Psychiatrie, Psychotherapie und Psychosomatik, Universität Augsburg | <https://www.bezirkskliniken-schwaben.de/kliniken/bezirkskrankenhaus-augsburg> |
| 4.     MPI für Psychiatrie | <https://www.mpg.de/153175/psychiatrie> |
| 5.     kbo Inn-Salzach-Klinikum Wasserburg | <https://kbo-isk.de/> |
| 6.     kbo-Lech-Mangfall-Klinik Garmisch Partenkirchen | <https://kbo-lmk.de/standorte/kbo-lech-mangfall-klinik-garmisch-partenkirchen> |
| 7.     kbo-Lech-Mangfall-Klinik Agatharied | <https://kbo-lmk.de/standorte/kbo-lech-mangfall-klinik-agatharied> |
| 8.     kbo-Lech-Mangfall-Klinik Landsberg am Lech | <https://kbo-lmk.de/standorte/kbo-lech-mangfall-klinik-landsberg-am-lech> |
| 9.     kbo Isar-Amper-Klinikum Taufkirchen/Vils | <https://kbo.de/kbo-isar-amper-klinikum> |
| 10.  kbo München Ost Haar | <https://kbo-iak.de/> |
| 11.  kbo-Isar-Amper-Klinikum München-Nord | <https://kbo-iak.de/standorte/kbo-klinik-fuer-psychiatrie-und-psychotherapie-nord-muenchen-schwabing> |
| 12.  kbo-Isar-Amper-Klinikum Fürstenfeldbruck | <https://kbo-iak.de/standorte/kbo-klinik-fuer-psychiatrie-und-psychotherapie-fuerstenfeldbruck> |
| 13.  BKH Günzburg, Universität Ulm | <https://www.uniklinik-ulm.de/psychiatrie-und-psychotherapie-ii.html> |
| 14.  BKH Kempten | <https://www.bezirkskliniken-schwaben.de/kliniken/bezirkskrankenhaus-kempten> |
| 15.  BKH Kaufbeuren | <https://www.bezirkskliniken-schwaben.de/kliniken/bezirkskrankenhaus-kaufbeuren> |
| 16.  BKH Memmingen | <https://www.bezirkskliniken-schwaben.de/kliniken/bezirkskrankenhaus-memmingen> |
| 17.  BKH Donauwörth | <https://www.bezirkskliniken-schwaben.de/kliniken/bezirkskrankenhaus-donauwoerth> |
| *Note.*  Links retrieved on 29/08/2022. | |

## ***1.2 Survey Structure***

*Key recommendations.* The four key recommendations were selected by the editors of the German schizophrenia guidelines based on their high-evidence and recommendation levels and practical importance (dose of antipsychotics, antipsychotics for relapse prevention, management of severe weight gain, application of cognitive behavioral therapy). In Supplementary Table 2, the selected recommendations are listed. Recommendation 1 corresponds to recommendation 22 in the current German guideline for schizophrenia (as of 2019), recommendation 2 corresponds to recommendation 36, recommendation 3 to 56 and recommendation 4 to 61 (DGPPN, 2019).

| Supplementary Table 2. The four selected key recommendations of the schizophrenia guideline. |
| --- |

| **Recommendation 1**  ***Dose of antipsychotics*** | Antipsychotics should be offered as low as possible and as high as necessary (lowest possible dosage) within the by international consensus recommended dosage range. Particularly in first-episode patients a low dose should be chosen as they are more sensitive to side effects and respond better to a lower dose. |
| --- | --- |
| **Recommendation 2 *Relapse prevention*** | People with schizophrenia (first-onset and multiple-onset) should be offered treatment with antipsychotics for relapse prevention after evaluating individual risk-benefit. |
| **Recommendation 3 *Severe weight gain*** | In cases of severe weight gain and the need to continue current antipsychotic medication, after implementation of psychotherapeutic and psychosocial interventions, treatment for weight loss should be offered by trying Metformin (first choice) or Topiramate (second choice) and by taking into account the risks for additional drug treatment. |
| **Recommendation 4 *Psychotherapy*** | People with schizophrenia should be offered cognitive behavioral therapy. |

*Living guideline.* In order to introduce the concept of a living guideline, the following description was presented to the participants:

*A Living Guideline can be understood as an optimized process of guideline development by continuously updating recommendations. In contrast to updating non-living guidelines, with a living guideline individual recommendations are updated rather than the whole guideline. The aim is to facilitate decision making through a timely adaptation of recommendations based on current evidence. Furthermore, a living guideline enables the involvement of users, patients and relatives in the guideline development process at any time. Digital, internet-based systems, such as the "MAGICapp" platform used for the SISYPHOS project, make the development of a living guideline possible. Living guidelines are usually updated once a year.*

*In the following, images of the "MAGICapp" are presented to illustrate a Living Guideline. We would like to ask you to look at them carefully, in order to answer questions about the format of Living Guidelines afterwards (e.g. with regard to clarity and comprehensibility).*

For an illustration of the living guideline, three screenshots of the unpublished living guideline for schizophrenia were presented. In order to give a broad overview, the table of contents, two examples of recommendations (social skills training and cognitive remediation) as well as a graphical comparison of two treatment options (e.g., to use for shared-decision making or to facilitate the decision making process) were shown. The images are presented under point 3 (questionnaire).

# **Supplementary Results**

## ***2.1 Participants’ characteristics***

Results indicate significant differences between excluded and included participants concerning gender, profession (medical doctors and other profession), setting (public hospital, research and other) and age, *p*s ≤ 0.032, see Supplementary Table 3. Moreover, subgroup comparisons indicate significant differences between professions regarding age, experience in the field of mental disorders and schizophrenia, as well as outpatient settings, *p*s ≤ 0.019 (see Supplementary Table 4). Between specialist doctors and assistant doctors differences were observed regarding workplace/setting (university hospital), age and experience (mental disorders in general and schizophrenia), *p*s ≤ 0.025 (see Supplementary Table 5). Significant differences between age-groups were found concerning profession (psychotherapists), professional settings (university hospital and practice within the framework of psychotherapy training) and experience (with schizophrenic disorders as well as mental disorders in general), *p*s ≤ 0.045 (see Supplementary Table 6).

| Supplementary Table 3. Descriptive characteristics of participants: Response comparisons between excluded and included participants. | | | | | | | | | | | | | | |
| --- | --- | --- | --- | --- | --- | --- | --- | --- | --- | --- | --- | --- | --- | --- |
|  |  |  |  |  |  |  | |  |  | **Test statistics** | | | | |
|  | **Included participants** | | | | **Excluded participants** | | | | |  | |  |  | |
|  |  | **N** | **%** |  |  | **N** | | **%** |  | **X²** | | **df** | **p** | |
| **Gender** |  |  |  |  |  |  | |  |  |  | |  |  | |
| Female |  | 439 | 68.1% |  |  | 85 | | 61.2% |  | 1.55 | | 1 | 0.213 | |
| Male |  | 439 | 31.9% |  |  | 85 | | 35.3% |  | 0.38 | | 1 | 0.540 | |
| Divers |  | 439 | 0.0% |  |  | 85 | | 3.5% |  | 15.58 | | 1 | <0.001 | |
| **Profession** | | | | | | |  | | | |  | | |  |
| Psychologist / psychotherapist |  | 439 | 18.2% |  |  | 85 | | 17.6% |  | 0.02 | | 1 | 0.009 | |
| Medical doctor |  | 439 | 42.6% |  |  | 85 | | 15.3% |  | 22.49 | | 1 | <0.001 | |
| Psychosocial therapist |  | 439 | 15.3% |  |  | 85 | | 20.0% |  | 1.19 | | 1 | 0.276 | |
| Caregiver |  | 439 | 21.9% |  |  | 85 | | 24.7% |  | 0.33 | | 1 | 0.565 | |
| Other |  | 439 | 2.1% |  |  | 85 | | 22.4% |  | 58.04 | | 1 | <0.001 | |
| **Setting^a^** |  |  |  |  |  |  | |  |  |  | |  |  | |
| **Inpatient** **setting** |  |  |  |  |  |  | |  |  |  | |  |  | |
| University hospital |  | 439 | 15.7% |  |  | 83 | | 16.9% |  | 0.07 | | 1 | 0.793 | |
| Public hospital |  | 439 | 72.9% |  |  | 83 | | 55.4% |  | 10.17 | | 1 | 0.001 | |
| Non-profit hospital |  | 439 | 6.4% |  |  | 83 | | 4.8% |  | 0.30 | | 1 | 0.587 | |
| Private hospital |  | 439 | 3.2% |  |  | 83 | | 2.4% |  | 0.14 | | 1 | 0.706 | |
| **Outpatient setting** |  |  |  |  |  |  | |  |  |  | |  |  | |
| Practice with health insurance license |  | 439 | 1.6% |  |  | 83 | | 3.6% |  | 1.52 | | 1 | 0.218 | |
| Private practice |  | 439 | 0.9% |  |  | 83 | | 0.0% |  | 0.76 | | 1 | 0.383 | |
| Practice within the framework of psychotherapy training |  | 439 | 2.3% |  |  | 83 | | 1.2% |  | 0.390 | | 1 | 0.532 | |
| **Research** |  | 439 | 2.3% |  |  | 83 | | 8.4% |  | 8.40 | | 1 | 0.004 | |
| **Other** |  | 439 | 0.9% |  |  | 83 | | 14.5% |  | 43.11 | | 1 | <0.001 | |
|  |  |  |  |  |  |  | |  |  |  | |  |  | |
|  |  | **N** | **M (SD)** |  |  | **N** | | **M (SD)** |  | **t** | | **df** | **p** | |
| **Age** |  |  |  |  |  |  | |  |  |  | |  |  | |
| Years |  | 439 | 41.41  (11.62) |  |  | 83 | | 37.71 (14.67) |  | -2.17 | | 102.31 | 0.032 | |
|  |  |  |  |  |  |  | |  |  |  | |  |  | |
|  |  |  | **Mdn**  **M (SD)** |  |  |  | | **Mdn**  **M (SD)** |  | **U** | | **Z** | **p** | |
| **Experience^b^** |  |  |  |  |  |  | |  |  |  | |  |  | |
| Mental disorders |  | 439 | 4.00  3.88 (0.91) |  |  | 61 | | 4.00  3.84 (0.99) |  | 12984.50 | | -0.40 | 0.688 | |
| Schizophrenic disorders |  | 439 | 3.00  3.43 (0.95) |  |  | 55 | | 3.00  3.44 (1.09) |  | 11476.00 | | -0.11 | 0.912 | |
| *Note.* *N* = number of participants, *M =* means, *SD* = standard deviations, *Mdn* = medians, *df* = degrees of freedom *X²* = Chi Square value, *t* = t-statistics, *U* = U-value (Mann-Whitney U-test), *Z* = Standard score. | | | | | | | | | | | | | | |

| Supplementary Table 4. *Descriptive characteristics of participants: Response comparisons between professions.* | | | | | | | | | | | | | | | | | | | | | | | | | | | | | | | | | | | | | | | | | | | | | | | | | | | | |
| --- | --- | --- | --- | --- | --- | --- | --- | --- | --- | --- | --- | --- | --- | --- | --- | --- | --- | --- | --- | --- | --- | --- | --- | --- | --- | --- | --- | --- | --- | --- | --- | --- | --- | --- | --- | --- | --- | --- | --- | --- | --- | --- | --- | --- | --- | --- | --- | --- | --- | --- | --- | --- |
|  | | | | | | |  | | | | | | | |  | | | | | | | | | |  | | | | | | | | | |  | | | | | | | | | **Test statistics** | | | | | | | | |
|  | | | | | | | **PSY**  N = 80 | | | | | | | | **MED**  N = 187 | | | | | | | | | | **PST**  N = 67 | | | | | | | | | | **CG**  N = 96 | | | | | | | | |  | | | | | | | | |
|  | | | | | | | **n** | | | **%** | | | | | **n** | | | | | **%** | | | | | **n** | | | | | **%** | | | | | **n** | | | | | **%** | | | | **Χ^2^** | | | **df** | | **p** | | | |
| **Gender** | | | | | | |  | | |  | | | | |  | | | | |  | | | | |  | | | | |  | | | | |  | | | | |  | | | |  | | |  | |  | | | |
| Female | | | | | | | 65 | | | 81.3% | | | | | 111 | | | | | 59.4% | | | | | 52 | | | | | 77.7% | | | | | 65 | | | | | 67.7% | | | | 15.75 | | | 3 | | <0.001 | | | |
| Male | | | | | | | 15 | | | 18.7% | | | | | 76 | | | | | 40.6% | | | | | 15 | | | | | 22.3% | | | | | 31 | | | | | 32.3% | | | |  | | |  | |  | | | |
| Divers | | | | | | | 0 | | | 0.0% | | | | | 0 | | | | | 0.0% | | | | | 0 | | | | | 0.0% | | | | | 0 | | | | | 0.0% | | | |  | | |  | |  | | | |
| **Setting^a^** | | | | | | |  | | |  | | | | |  | | | | |  | | | | |  | | | | |  | | | | |  | | | | |  | | | |  | | |  | |  | | | |
| **Inpatient** **setting** | | | | | | |  | | |  | | | | |  | | | | |  | | | | |  | | | | |  | | | | |  | | | | |  | | | |  | | |  | |  | | | |
| University hospital | | | | | | | 12 | | | 15.0% | | | | | 38 | | | | | 20.3% | | | | | 8 | | | | | 11.9% | | | | | 11 | | | | | 11.5% | | | | 4.94 | | | 3 | | 0.176 | | | |
| Public hospital | | | | | | | 58 | | | 72.5% | | | | | 130 | | | | | 69.5% | | | | | 49 | | | | | 73.1% | | | | | 75 | | | | | 78.1% | | | | 2.37 | | | 3 | | 0.499 | | | |
| Non-profit hospital | | | | | | | 2 | | | 2.5% | | | | | 9 | | | | | 5.3% | | | | | 8 | | | | | 11.9% | | | | | 8 | | | | | 8.3% | | | | 6.30 | | | 3 | | 0.098 | | | |
| Private hospital | | | | | | | 3 | | | 3.8% | | | | | 5 | | | | | 2.7% | | | | | 3 | | | | | 4.5% | | | | | 3 | | | | | 3.1% | | | | 0.59 | | | 3 | | 0.873^b^ | | | |
| **Outpatient setting** | | | | | | |  | | |  | | | | |  | | | | |  | | | | |  | | | | |  | | | | |  | | | | |  | | | |  | | |  | |  | | | |
| Practice with health insurance license | | | | | | | 2 | | | 2.5% | | | | | 4 | | | | | 2.1% | | | | | 0 | | | | | 0.0% | | | | | 0 | | | | | 0.0% | | | | 3.77 | | | 3 | | 0.334^b^ | | | |
| Private practice | | | | | | | 2 | | | 2.5% | | | | | 0 | | | | | 0.0% | | | | | 2 | | | | | 3.0% | | | | | 0 | | | | | 0.0% | | | | 7.87 | | | 3 | | 0.019^b^ | | | |
| Practice within the framework of psychotherapy training | | | | | | | 7 | | | 8.8% | | | | | 3 | | | | | 1.6% | | | | | 0 | | | | | 0.0% | | | | | 0 | | | | | 0.0% | | | | 18.85 | | | 3 | | 0.001^b^ | | | |
| **Research** | | | | | | | 3 | | | 3.8% | | | | | 4 | | | | | 2.1% | | | | | 1 | | | | | 1.5% | | | | | 1 | | | | | 1.0% | | | | 1.710 | | | 3 | | 0.671^b^ | | | |
| **Other** | | | | | | | 1 | | | 1.3% | | | | | 2 | | | | | 1.1% | | | | | 1 | | | | | 1.5% | | | | | 0 | | | | | 0.0% | | | | 1.26 | | | 3 | | 0.683^b^ | | | |
|  | | |  | | | |  | | | | | | | |  | | | | | | | | | |  | | | | | | | | | |  | | | | | | | | |  | | | | | | | | |
|  | | |  | | | | **PSY**  N = 80 | | | | | | | | | **MED**  N = 187 | | | | | | | | | | | **PST**  N = 67 | | | | | | | | | **CG**  N = 96 | | | | | | | |  | | | | | | | | |
|  | | |  | |  | | **M** | | | | **SD** | | | | | **M** | | | | | | **SD** | | | | | **M** | | | | **SD** | | | | | **M** | | | | | **SD** | | | **Welch’s**  **F^c^** | | | | **df** | | **p** | | |
| **Age** | | |  | |  | |  | | | |  | | | | |  | | | | | |  | | | | |  | | | |  | | | | |  | | | | |  | | |  | | | |  | |  | | |
| Years | | |  | |  | | 34.49 | | | | 9.92 | | | | | 42.66 | | | | | | 10.88 | | | | | 45.24 | | | | 11.29 | | | | | 41.95 | | | | | 12.60 | | | 16.16 | | | | 3 | | <0.001 | | |
|  | | |  | |  | |  | | | |  | | | | |  | | | | | |  | | | | |  | | | |  | | | | |  | | | | |  | | |  | | | |  | |  | | |
|  | | |  | |  | | **Mdn** | | | | **M (SD)** | | | | | **Mdn** | | | | | | **M (SD)** | | | | | **Mdn** | | | | **M (SD)** | | | | | **Mdn** | | | | | **M (SD)** | | | **H** | | | | **df** | | **p** | | |
| **Experience^d^** | | | | | |  | | | | | | |  | | | | |  | | | | | |  | | | | | | | |  | | | | | |  | | | | | |  | | | |  | |  | | |
| Mental disorders | | |  | |  | | 3.00 | | | | 3.45 (0.93) | | | | | 4.00 | | | | | | 3.91 (0.90) | | | | | 4.00 | | | | 4.00 (0.84) | | | | | 4.00 | | | | | 4.13 (0.84) | | | 25.38 | | | | 3 | | <0.001 | | |
| Schizophrenic disorders | | |  | |  | | 3.00 | | | | 2.79 (0.82) | | | | | 4.00 | | | | | | 3.51 (0.91) | | | | | 3.00 | | | | 3.41 (0.94) | | | | | 4.00 | | | | | 3.78  (0.92) | | | 46.07 | | | | 3 | | <0.001 | | |
|  | | **Subgroup analyses:**  **Chi Square Test (*p*-values Bonferroni corrected)** | | | | | | | | | | | | | | | | | | | | | | | | | | | | | | | | | | | | | | | | | | | | | | | | | | |
|  | | **PSY _[vs]_ PST** | | | | | | | | | | **PSY _[vs]_ MED** | | | | | | | | | **PSY _[vs]_ CG** | | | | | | | | **PST _[vs]_ MED** | | | | | | | | | | **PST _[vs]_ CG** | | | | | | | **MED _[vs]_ CG** | | | | | | |
|  | | **X²** | | | | | | **p** | | | | **X²** | | | | | **p** | | | | **X²** | | | | | **p** | | | **X²** | | | | | **p** | | | | | **X²** | | | | **p** | | | **X²** | | | | | | **p** |
| **Setting^a^** | |  | | | | | |  | | | |  | | | | |  | | | |  | | | | |  | | |  | | | | |  | | | | |  | | | |  | | |  | | | | | |  |
| Private practice | | 0.03 | | | | | | 1.000^b^ | | | | 4.71 | | | | | 0.534^b^ | | | | 2.43 | | | | | 1.000^b^ | | | 5.63 | | | | | 0.414^b^ | | | | | 2.90 | | | | 1.000^b^ | | | - | | | | | | - |
| Practice within the framework of psychotherapy training | | 6.16 | | | | | | 0.096^b^ | | | | 7.94 | | | | | 0.054^b^ | | | | 8.75 | | | | | 0.018^b^ | | | 1.09 | | | | | 1.000^b^ | | | | | - | | | | - | | | 1.56 | | | | | | 1.000^b^ |
|  | **Subgroup analyses:**  **Bonferroni Test (*p*-values Bonferroni corrected)** | | | | | | | | | | | | | | | | | | | | | | | | | | | | | | | | | | | | | | | | | | | | | | | | | | | |
|  | **PSY _[vs]_ PST** | | | | | | | | **PSY _[vs]_ MED** | | | | | | | | | | **PSY _[vs]_ CG** | | | | | | | | | **PST _[vs]_ MED** | | | | | | | | | **PST _[vs]_ CG** | | | | | | | | **MED _[vs]_ CG** | | | | | | | |
|  | **[I-J]** | | | | | | | | **[I-J]** | | | | | | | | | | **[I-J]** | | | | | | | | | **[I-J]** | | | | | | | | | **[I-J]** | | | | | | | | **[I-J]** | | | | | | | |
|  | **95% CI** | | | | | | | | **95% CI** | | | | | | | | | | **95% CI** | | | | | | | | | **95% CI** | | | | | | | | | **95% CI** | | | | | | | | **95% CI** | | | | | | | |
|  | **p** | | | | | | | | **p** | | | | | | | | | | **p** | | | | | | | | | **p** | | | | | | | | | **p** | | | | | | | | **p** | | | | | | | |
| **Age** |  | | | | | | | |  | | | | | | | | | |  | | | | | | | | |  | | | | | | | | |  | | | | | | | |  | | | | | | | |
|  | -10.75 | | | | | | | | -8.17 | | | | | | | | | | -7.46 | | | | | | | | | -2.58 | | | | | | | | | 3.29 | | | | | | | | 0.71 | | | | | | | |
|  | [-15.7;-5.8] | | | | | | | | [-12.1;-4.2] | | | | | | | | | | [-12.0;-3.0] | | | | | | | | | [-6.8;1.6] | | | | | | | | | [-1.4;8.0] | | | | | | | | [-3.0;4.4] | | | | | | | |
|  | <0.001 | | | | | | | | <0.001 | | | | | | | | | | <0.001 | | | | | | | | | 0.634 | | | | | | | | | 0.391 | | | | | | | | 1.000 | | | | | | | |
|  | **Subgroup analyses:**  **Dunn-Bonferroni-Test (*p*-values Bonferroni corrected)** | | | | | | | | | | | | | | | | | | | | | | | | | | | | | | | | | | | | | | | | | | | | | | | | | | | |
|  | **PSY _[vs]_ PST** | | | | | | | | **PSY _[vs]_ MED** | | | | | | | | | | **PSY _[vs]_ CG** | | | | | | | | | **PST _[vs]_ MED** | | | | | | | | | **PST _[vs]_ CG** | | | | | | | | **MED _[vs]_ CG** | | | | | | | |
|  | **Z** | | | **p** | | | | | **Z** | | | | | **p** | | | | | **Z** | | | | **p** | | | | | **Z** | | | | | **p** | | | | **Z** | | | | | **p** | | | **Z** | | | | | | **p** | |
| **Experience^d^** | | | | | | | | | | | | | | | | | | | | | | | | | | | | | | | | | | | | | | | | | | | | | | | | | | | | |
| Mental disorders | -3.51 | | | 0.003 | | | | | -3.80 | | | | | 0.001 | | | | | -4.84 | | | | <0.001 | | | | | -0.51 | | | | | 1.000 | | | | 0.95 | | | | | 1.000 | | | -1.79 | | | | | | 0.441 | |
| Schizo-phrenic disorders | -3.69 | | | 0.001 | | | | | -5.42 | | | | | <0.001 | | | | | -6.58 | | | | <0.001 | | | | | 0.86 | | | | | 1.000 | | | | 2.52 | | | | | 0.070 | | | -2.23 | | | | | | 0.156 | |
| *Note.* ^a^Multiple answers were posssible. ^b^Due to expected cell counts less than 5 in more than 20% of cells, fisher’s exact test was conducted. ^c^Welch’s analysis of variance was performed due to inequality of variances. ^d^Participants were asked how they would rate their experience in treating people with mental disorders or schizophrenic disorders (1 = not at all experienced – 5 = very experienced). *N* = number of participants. *M* = means. *SD* = standard deviations. *Mdn* = medians. *I-J* = difference in mean between groups. *95% CI* = 95% confidence interval for the difference in means. *H =* H-value. *Z* = Standard score. PSY = psychologists/psychotherapists, MED = medical doctors, PST = psychosocial therapists, CG = caregivers. | | | | | | | | | | | | | | | | | | | | | | | | | | | | | | | | | | | | | | | | | | | | | | | | | | | | |

| Supplementary Table 5. Descriptive characteristics of participants: Response comparisons between specialist and assistant doctors. | | | | | | | | | | | |
| --- | --- | --- | --- | --- | --- | --- | --- | --- | --- | --- | --- |
|  |  |  |  |  |  |  |  |  | **Chi Square Test** | | |
|  | **Specialist doctors**  N = 104 | | | | **Assistant doctors**  N = 83 | | | |  |  |  |
|  |  | **n** | **%Yes** |  |  | **n** | **%Yes** |  | **X²** | **df** | **p** |
| **Gender** |  |  |  |  |  |  |  |  |  |  |  |
| Female |  | 63 | 60.6% |  |  | 48 | 57.8% |  | 0.144 | 1 | 0.704 |
| Male |  | 41 | 39.4% |  |  | 35 | 42.2% |  |  |  |  |
| Divers |  | 0 | 0.0% |  |  | 0 | 0.0% |  |  |  |  |
| **Setting^a^** |  |  |  |  |  |  |  |  |  |  |  |
| **Inpatient** **setting** |  |  |  |  |  |  |  |  |  |  |  |
| University hospital |  | 15 | 14.4% |  |  | 23 | 27.7% |  | 5.03 | 1 | 0.025 |
| Public hospital |  | 76 | 73.1% |  |  | 54 | 65.1% |  | 1.40 | 1 | 0.237 |
| Non-profit hospital |  | 6 | 5.8% |  |  | 4 | 4.8% |  | 0.08 | 1 | 1.000^b^ |
| Private hospital |  | 4 | 3.8% |  |  | 1 | 1.2% |  | 1.24 | 1 | 0.384^b^ |
| **Outpatient setting** |  |  |  |  |  |  |  |  |  |  |  |
| Practice with health insurance license |  | 4 | 3.8% |  |  | 0 | 0.0% |  | 3.26 | 1 | 0.130^b^ |
| Private practice |  | 0 | 0.0% |  |  | 0 | 0.0% |  | - | - | - |
| Practice within the framework of psychotherapy training |  | 0 | 0.0% |  |  | 3 | 3.6% |  | 3.820 | 1 | 0.086^b^ |
| **Research** |  | 2 | 1.9% |  |  | 2 | 2.4% |  | 0.05 | 1 | 1.000^b^ |
| **Other** |  | 0 | 0.0% |  |  | 2 | 2.4% |  | 2.53 | 1 | 0.196^b^ |
|  |  |  |  |  |  |  |  |  |  |  |  |
|  |  | **M** | **SD** |  |  | **M** | **SD** |  | **t** | **df** | **p** |
| **Age** |  |  |  |  |  |  |  |  |  |  |  |
| Years |  | 48.78 | 9.05 |  |  | 34.99 | 7.67 |  | 11.28 | 184.31 | <0.001 |
|  |  |  |  |  |  |  |  |  |  |  |  |
|  |  | **Mdn** | **M (SD)** |  |  | **Mdn** | **M (SD)** |  | **U** | **Z** | **p** |
| **Experience^c^** |  |  |  |  |  |  |  |  |  |  |  |
| Mental disorders |  | 4.00 | 4.40 (0.60) |  |  | 3.00 | 3.30 (0.84) |  | 1407.00 | -8.38 | <0.001 |
| Schizophrenic disorders |  | 4.00 | 3.94 (0.75) |  |  | 3.00 | 2.95 (0.80) |  | 1724.00 | -7.36 | <0.001 |
| *Note.* ^a^Multiple answers were posssible. ^b^Due to expected cell counts less than 5 in more than 20% of cells, fisher’s exact test was conducted. ^c^Participants were asked how they would rate their experience in treating people with mental disorders or schizophrenic disorders (1 = not at all experienced – 5 = very experienced). *N* = number of participants, *M =* means, *SD* = standard deviations, *Mdn* = medians, *df* = degrees of freedom *X²* = Chi Square value, *t* = t-statistics, *U* = U-value (Mann-Whitney U-test), *Z* = Standard score. | | | | | | | | | | | |

| Supplementary Table 6. *Descriptive characteristics of participants: Response comparisons between age groups.* | | | | | | | | | |
| --- | --- | --- | --- | --- | --- | --- | --- | --- | --- |
|  |  | |  | |  | | **Test statistics** | | |
|  | **Young**  **(20 – 34 years)**  N = 163 | | **Middle-aged**  **(35 – 49 years)**  N = 144 | | **Older**  **(50 – 66 years)**  N = 132 | |  | | |
|  | **n** | **%Yes** | **n** | **%Yes** | **n** | **%Yes** | **Χ^2^** | **df** | **p** |
| **Gender** |  |  |  |  |  |  |  |  |  |
| Female | 115 | 70.6% | 100 | 69.4% | 84 | 63.6% | 1.78 | 2 | 0.410 |
| Male | 48 | 29.4% | 44 | 30.6% | 48 | 36.4% |  |  |  |
| Divers | 0 | 0.0% | 0 | 0.0% | 0 | 0.0% |  |  |  |
| **Profession** |  |  |  | 0.0% |  | 0.0% |  |  |  |
| Psychologist / psychotherapist | 54 | 33.1% | 16 | 11.1% | 10 | 7.6% | 39.23 | 2 | <0.001 |
| Medical doctor | 63 | 38.7% | 69 | 47.9% | 55 | 41.7% | 2.75 | 2 | 0.253 |
| Psychosocial therapist | 32 | 19.6% | 30 | 20.8% | 34 | 25.8% | 1.74 | 2 | 0.420 |
| Caregiver | 11 | 6.7% | 25 | 17.4% | 31 | 23.5% | 16.53 | 2 | <0.001 |
| Else | 3 | 1.8% | 4 | 2.8% | 2 | 1.5% | 0.64 | 2 | 0.767^b^ |
| **Setting^a^** |  |  |  |  |  |  |  |  |  |
| **Inpatient** **setting** |  |  |  |  |  |  |  |  |  |
| University hospital | 32 | 19.6% | 13 | 9.0% | 24 | 18.2% | 7.36 | 2 | 0.025 |
| Public hospital | 108 | 66.3% | 113 | 78.5% | 99 | 75.0% | 6.20 | 2 | 0.045 |
| Non-profit hospital | 9 | 5.5% | 12 | 8.3% | 7 | 5.3% | 1.38 | 2 | 0.502 |
| Private hospital | 8 | 4.9% | 4 | 2.8% | 2 | 1.5% | 2.59 | 2 | 0.247^b^ |
| **Outpatient setting** |  |  |  |  |  |  |  |  |  |
| Practice with health insurance license | 2 | 1.2% | 0 | 0.0% | 5 | 3.8% | 5.73 | 2 | 0.034^b^ |
| Private practice | 1 | 0.6% | 1 | 0.7% | 2 | 1.5% | 0.91 | 2 | 0.690^b^ |
| Practice within the framework of psychotherapy training | 7 | 4.3% | 3 | 2.1% | 0 | 0.0% | 6.15 | 2 | 0.039^b^ |
| **Research** | 7 | 4.3% | 2 | 1.4% | 1 | 0.8% | 4.10 | 2 | 0.125^b^ |
| **Other** | 3 | 1.8% | 1 | 0.7% | 0 | 0.0% | 2.29 | 2 | 0.394^b^ |
|  |  | |  | |  | |  | | |
|  | **Mdn** | **M (SD)** | **Mdn** | **M (SD)** | **Mdn** | **M (SD)** | **H** | **df** | **p** |
| **Experience^c^** |  |  |  |  |  |  |  |  |  |
| Mental disorders | 3.00 | 3.18 (0.81) | 4.00 | 4.16 (0.70) | 5.00 | 4.44 (0.66) | 155.59 | 2 | <0.001 |
| Schizophrenic disorders | 3.00 | 2.84 (0.81) | 4.00 | 3.58 (0.81) | 4.00 | 3.98 (0.85) | 104.10 | 2 | <0.001 |
|  |  |  |  |  |  |  |  |  |  |
|  | **Subgroup analyses:**  **Chi Square Test (*p*-values Bonferroni corrected)** | | | | | |  |  |  |
|  | **Young**  **(20 – 34 years)**  **[vs]**  **Older**  **(50 – 66 years)** | | **Middle-aged**  **(35 – 49 years) [vs]**  **Older**  **(50 – 66 years)** | | **Young**  **(20 – 34 years)**  **[vs]**  **Middle-aged**  **(35 – 49 years)** | |  |  |  |
|  | **X²** | **p** | **X²** | **p** | **X²** | **p** |  |  |  |
| **Profession** |  |  |  |  |  |  |  |  |  |
| Psychologist / psychotherapist | 28.03 | <0.001 | 1.009 | 0.945 | 21.56 | <0.001 |  |  |  |
| Caregiver | 1.58 | 0.627 | 0.94 | 0.999 | 0.07 | 1.000 |  |  |  |
| **Setting^a^** |  |  |  |  |  |  |  |  |  |
| **Inpatient** **setting** |  |  |  |  |  |  |  |  |  |
| University hospital | 0.10 | 1.000 | 4.97 | 0.078 | 6.87 | 0.027 |  |  |  |
| Public hospital | 2.66 | 0.309 | 0.47 | 1.000 | 5.66 | 0.051 |  |  |  |
| **Outpatient setting** |  |  |  |  |  |  |  |  |  |
| Practice with health insurance license | 2.07 | 0.747^b^ | 5.56 | 0.072^b^ | 1.78 | 1.000^b^ |  |  |  |
| Practice within the framework of psychotherapy training | 5.81 | 0.054^b^ | 2.78 | 0.747^b^ | 1.19 | 1.000^b^ |  |  |  |
|  |  |  |  |  |  |  |  |  |  |
|  | **Subgroup analyses:**  **Dunn-Bonferroni-Test (*p*-values Bonferroni corrected)** | | | | | |  |  |  |
|  | **Young**  **(20 – 34 years)**  **[vs]**  **Older**  **(50 – 66 years)** | | **Middle-aged**  **(35 – 49 years) [vs]**  **Older**  **(50 – 66 years)** | | **Young**  **(20 – 34 years)**  **[vs]**  **Middle-aged**  **(35 – 49 years)** | |  |  |  |
|  | **Z** | **p** | **Z** | **p** | **Z** | p |  |  |  |
| **Experience^c^** |  |  |  |  |  |  |  |  |  |
| Mental disorders | -11.75 | <0.001 | -40.32 | <0.001 | -9.11 | <0.001 |  |  |  |
| Schizophrenic disorders | -9.97 | <0.001 | -3.45 | 0.001 | -9.97 | <0.001 |  |  |  |
| *Note.* ^a^Multiple answers were posssible. ^b^Due to expected cell counts less than 5 in more than 20% of cells, fisher’s exact test was conducted. ^c^Participants were asked how they would rate their experience in treating people with mental disorders or schizophrenic disorders (1 = not at all experienced – 5 = very experienced). *N* = number of participants, *M =* means, *SD* = standard deviations, *Mdn* = medians, *df* = degrees of freedom *X²* = Chi Square value. *H =* H-value. | | | | | | | | | |

## ***2.2 Awareness, agreement, adoption and adherence: Implementation status of the guideline for schizophrenia and its key recommendations***

| Supplementary Table 7. *Subgroup analyses of Mean response comparisons between professions regarding the implementation status (awareness, agreement, adoption and adherence) of the guideline schizophrenia in general as well as of four selected recommendations.* | | | | | | | | | | | | | | | | | | | | | | | | | | | | | |
| --- | --- | --- | --- | --- | --- | --- | --- | --- | --- | --- | --- | --- | --- | --- | --- | --- | --- | --- | --- | --- | --- | --- | --- | --- | --- | --- | --- | --- | --- |
|  | | **Chi Square Test (*p*-values Bonferroni corrected)** | | | | | | | | | | | | | | | | | | | | | | | | | | | |
|  | **PSY _[vs]_ PST** | | | | | **PSY_[vs]_MED** | | | | **PSY _[vs]_ CG** | | | | | **PST _[vs]_ MED** | | | | | **PST _[vs]_ CG** | | | | | **MED _[vs]_ CG** | | | | |
|  |  | | **Χ^2^** | **p** |  |  | **Χ^2^** | **p** |  | |  | **Χ^2^** | **p** |  | |  | **Χ^2^** | **p** |  | |  | **Χ^2^** | **p** |  | |  | **Χ^2^** | **p** |  |
| Awareness | | | | | | | | | | |  |  |  |  | |  |  |  |  | |  |  |  |  | |  |  |  |  |
| Guideline for schizophrenia [Q13] |  | | 0.04 | 1.000 |  |  | 25.19 | <0.001 |  | |  | 1.95 | 0.978 |  | |  | 23.98 | <0.001 |  | |  | 2.30 | 0.774 |  | |  | 14.49 | <0.001 |  |
| Recommendation 1 [Q17] |  | | 3.55 | 0.354 |  |  | 37.52 | <0.001 |  | |  | 5.85 | 0.096 |  | |  | 66.21 | <0.001 |  | |  | 17.49 | <0.001 |  | |  | 10.32 | 0.006 |  |
| Recommendation 2 [Q21] |  | | 6.68 | 0.060 |  |  | 19.10 | <0.001 |  | |  | 0.35 | 1.000 |  | |  | 54.85 | <0.001 |  | |  | 9.79 | 0.012 |  | |  | 12.99 | 0.006 |  |
| Recommendation 3 [Q25]  Recommendation 4 [Q29] |  | | 0.21  19.25 | 1.000  <0.001 |  |  | 44.78  0.44 | <0.001  1.000 |  | |  | 5.64  17.03 | 0.108  <0.001 |  | |  | 30.75  23.78 | <0.001  <0.001 |  | |  | 2.93  0.28 | 0.522  1.000 |  | |  | 18.72  21.64 | <0.001  <0.001 |  |
| Agreement | | | | | | | | | | |  |  |  |  | |  |  |  |  | |  |  |  |  | |  |  |  |  |
| Guideline for schizophrenia [Q14] |  | | 3.45 | 0.378 |  |  | 25.31 | <0.001 |  | |  | 0.52 | 1.000 |  | |  | 43.30 | <0.001 |  | |  | 1.58 | 1.000 |  | |  | 37.18 | <0.001 |  |
| Recommendation 1 [Q18] |  | | 10.58 | 0.006 |  |  | 7.32 | 0.042 |  | |  | 1.49 | 1.000 |  | |  | 46.72 | <0.001 |  | |  | 4.95 | 0.156 |  | |  | 18.80 | <0.001 |  |
| Recommendation 2 [Q22] |  | | 20.15 | <0.001 |  |  | 0.99 | 1.000 |  | |  | 1.96 | 1.000 |  | |  | 49.35 | <0.001 |  | |  | 11.12 | 0.006 |  | |  | 7.90 | 0.030 |  |
| Recommendation 3 [Q26]  Recommendation 4 [Q30] |  | | 0.10  20.05 | 1.000  <0.001 |  |  | 22.47  1.65 | <0.001  1.000 |  | |  | 1.18  10.35 | 1.000  0.006 |  | |  | 19.87  19.66 | <0.001  <0.001 |  | |  | 1.63  2.28 | 1.000  0.786 |  | |  | 12.76  7.70 | 0.006  0.036 |  |
| Adoption | | | | | | | | | | |  |  |  |  | |  |  |  |  | |  |  |  |  | |  |  |  |  |
| Guideline for schizophrenia [Q15] |  | | 0.48 | 1.000 |  |  | 3.90 | 0.288 |  | |  | 0.22 | 1.000 |  | |  | 5.63 | 0.108 |  | |  | 0.10 | 1.000 |  | |  | 6.94 | 0.048 |  |
| Recommendation 1 [Q19] |  | | 5.63 | 0.108 |  |  | 3.98 | 0.276 |  | |  | 0.13 | 1.000 |  | |  | 22.61 | <0.001 |  | |  | 4.41 | 0.216 |  | |  | 6.32 | 0.072 |  |
| Recommendation 2 [Q23] |  | | 5.42 | 0.120 |  |  | 14.07 | 0.001 |  | |  | 0.12 | 1.000 |  | |  | 40.86 | <0.001 |  | |  | 4.01 | 0.270 |  | |  | 17.62 | <0.001 |  |
| Recommendation 3 [Q27]  Recommendation 4 [Q31] |  | | 2.91  8.93 | 0.528  0.018 |  |  | 11.18  15.98 | 0.006  <0.001 |  | |  | 0.12  12.19 | 1.000  0.006 |  | |  | 20.73  0.06 | <0.001  1.000 |  | |  | 3.95  0.06 | 0.282  1.000 |  | |  | 8.57  0.004 | 0.018  1.000 |  |
| Adherence | | | | | | | | | | |  |  |  |  | |  |  |  |  | |  |  |  |  | |  |  |  |  |
| Guideline for schizophrenia [Q16] |  | | 2.23 | 0.810 |  |  | 0.02 | 1.000 |  | |  | 0.13 | 1.000 |  | |  | 2.44 | 0.708 |  | |  | 1.69 | 1.000 |  | |  | 0.30 | 1.000 |  |
| Recommendation 1 [Q20] |  | | 1.07 | 1.000 |  |  | 0.01 | 1.000 |  | |  | 1.76 | 1.000 |  | |  | 1.58 | 1.000 |  | |  | 0.00 | 1.000 |  | |  | 2.94 | 0.516 |  |
| Recommendation 2 [Q24] |  | | 4.80 | 0.174 |  |  | 3.25 | 0.426 |  | |  | 0.08 | 1.000 |  | |  | 13.92 | 0.001 |  | |  | 3.92 | 0.288 |  | |  | 4.57 | 0.198 |  |
| Recommendation 3 [Q28]  Recommendation 4 [Q32] |  | | 0.36  0.00 | 1.000  1.000 |  |  | 0.14  2.38 | 1.000  0.738 |  | |  | 0.02  0.34 | 1.000  1.000 |  | |  | 0.16  1.68 | 1.000  1.000 |  | |  | 0.59  0.31 | 1.000  1.000 |  | |  | 0.34  0.39 | 1.000  1.000 |  |
| *Note.* *X²* = Chi Square value. Numbers of questions are displayed in square brackets. The complete questionnaire is shown under point 3. Total = all participants included, PSY = psychologists/psychotherapists, MED = medical doctors, PST = psychosocial therapists, CG = caregivers. | | | | | | | | | | | | | | | | | | | | | | | | | | | | | |
| Recommendation 1: *Antipsychotics should be offered as low as possible and as high as necessary (lowest possible dosage) within the by international consensus recommended dosage range. Particularly in first-episode patients a low dose should be chosen as they are more sensitive to side effects and respond better to a lower dose.* Recommendation 2: *People with schizophrenia (first-onset and multiple-onset) should be offered treatment with antipsychotics for relapse prevention after evaluating individual risk-benefit.* Recommendation 3: *In cases of severe weight gain and the need to continue current antipsychotic medication, after implementation of psychotherapeutic and psychosocial interventions, treatment for weight loss should be offered by trying Metformin (first choice) or Topiramate (second choice) and by taking into account the risks for additional drug treatment.* Recommendation 4: *People with schizophrenia should be offered cognitive behavioral therapy.* | | | | | | | | | | | | | | | | | | | | | | | | | | | | | |

| Supplementary Table 8. Mean response comparisons between specialist and assistant doctors regarding the implementation status (awareness, agreement, adoption and adherence) of the guideline schizophrenia in general as well as of four selected recommendations. | | | | | | | | | | | |
| --- | --- | --- | --- | --- | --- | --- | --- | --- | --- | --- | --- |
|  |  |  |  |  |  |  |  |  | **Chi Square Test** | | |
|  | **Specialist Doctor** | | | | **Assistant Doctor** | | | |  |  |  |
|  |  | **N** | **%Yes** |  |  | **N** | **%Yes** |  | **X²** | **df** | **p** |
| Awareness |  |  |  |  |  |  |  |  |  |  |  |
| Guideline for schizophrenia [Q13] |  | 104 | 71.2% |  |  | 83 | 39.8% |  | 18.59 | 1 | <0.001 |
| Recommendation 1 [Q17] |  | 100 | 98.0% |  |  | 82 | 90.2% |  | 5.22 | 1 | 0.022 |
| Recommendation 2 [Q21] |  | 100 | 97.0% |  |  | 79 | 91.1% |  | 2.87 | 1 | 0.090 |
| Recommendation 3 [Q25] |  | 98 | 59.2% |  |  | 45 | 59.2% |  | 0.00 | 1 | 0.997 |
| Recommendation 4 [Q32] |  | 98 | 95.9% |  |  | 75 | 72.0% |  | 19.66 | 1 | <0.001 |
| Agreement |  |  |  |  |  |  |  |  |  |  |  |
| Guideline for schizophrenia [Q14] |  | 104 | 77.9% |  |  | 83 | 48.2% |  | 17.82 | 1 | <0.001 |
| Recommendation 1 [Q18] |  | 100 | 99.0% |  |  | 82 | 93.8% |  | 3.67 | 1 | 0.055 |
| Recommendation 2 [Q22] |  | 100 | 97.0% |  |  | 79 | 94.9% |  | 0.50 | 1 | 0.479 |
| Recommendation 3 [Q26] |  | 98 | 44.9% |  |  | 76 | 61.8% |  | 4.93 | 1 | 0.026 |
| Recommendation 4 [Q30] |  | 98 | 93.9% |  |  | 75 | 78.7% |  | 8.86 | 1 | 0.003 |
| Adoption |  |  |  |  |  |  |  |  |  |  |  |
| Guideline for schizophrenia [Q15] |  | 98 | 75.5% |  |  | 68 | 52.9% |  | 9.15 | 1 | 0.002 |
| Recommendation 1 [Q19] |  | 100 | 85.0% |  |  | 82 | 75.6% |  | 2.56 | 1 | 0.110 |
| Recommendation 2 [Q23] |  | 100 | 92.0% |  |  | 79 | 83.5% |  | 3.05 | 1 | 0.081 |
| Recommendation 3 [Q27] |  | 98 | 38.8% |  |  | 76 | 56.6% |  | 5.45 | 1 | 0.020 |
| Recommendation 4 [Q31] |  | 97 | 54.6% |  |  | 75 | 46.7% |  | 1.08 | 1 | 0.300 |
| Adherence |  |  |  |  |  |  |  |  |  |  |  |
| Guideline for schizophrenia [Q16] |  | 95 | 11.6% |  |  | 60 | 3.3% |  | 3.25 | 1 | 0.071 |
| Recommendation 22 [Q20] |  | 93 | 29.0% |  |  | 72 | 23.6% |  | 0.61 | 1 | 0.435 |
| Recommendation 36 [Q24] |  | 94 | 44.7% |  |  | 68 | 52.9% |  | 1.08 | 1 | 0.299 |
| Recommendation 56 [Q28] |  | 80 | 1.3% |  |  | 58 | 10.3% |  | 5.78 | 1 | 0.016 |
| Recommendation 61 [Q32] |  | 92 | 3.3% |  |  | 60 | 6.7% |  | 0.96 | 1 | 0.327 |
| *Note.* *N* = number of participants, *df* = degrees of freedom *X²* = Chi Square value. Numbers of questions are displayed in square brackets. The complete questionnaire is shown under point 3. | | | | | | | | | | | |
| Recommendation 22: *Antipsychotics should be offered as low as possible and as high as necessary (lowest possible dosage) within the by international consensus recommended dosage range. Particularly in first-episode patients a low dose should be chosen as they are more sensitive to side effects and respond better to a lower dose.* Recommendation 36: *People with schizophrenia (first-onset and multiple-onset) should be offered treatment with antipsychotics for relapse prevention after evaluating individual risk-benefit.* Recommendation 56: *In cases of severe weight gain and the need to continue current antipsychotic medication, after implementation of psychotherapeutic and psychosocial interventions, treatment for weight loss should be offered by trying Metformin (first choice) or Topiramate (second choice) and by taking into account the risks for additional drug treatment.* Recommendation 61: *People with schizophrenia should be offered cognitive behavioral therapy.* | | | | | | | | | | | |

## ***2.3 Attitude towards the living guideline for schizophrenia***

| Supplementary Table 9. *Subgroup comparisons between age groups: Attitude towards the living guideline for schizophrenia.* | | | | | | | | | | |
| --- | --- | --- | --- | --- | --- | --- | --- | --- | --- | --- |
|  | **Subgroup analyses: Dunn-Bonferroni-Test**  **(*p*-values Bonferroni corrected)** | | | | | | | | | |
|  |  | **Young**  **(20 – 34 years)**  **[vs]**  **Older**  **(50 – 66 years)** | |  | **Middle-aged**  **(35 – 49 years) [vs]**  **Older**  **(50 – 66 years)** | |  | **Young**  **(20 – 34 years)**  **[vs]**  **Middle-aged**  **(35 – 49 years)** | |  |
|  |  | **Z** | **p** |  | **Z** | **p** |  | **Z** | **p** |  |
| User-friendliness |  |  |  |  |  |  |  |  |  |  |
| I find the layout appealing and the content clearly presented. [Q33] |  | 3.09 | 0.006 |  | 1.64 | 0.302 |  | 1.41 | 0.476 |  |
| I can imagine getting along well with the Living Guideline. [Q34] |  | 3.36 | 0.001 |  | 1.11 | 0.800 |  | 2.25 | 0.074 |  |
| The Living Guideline seems clearer than the previous print version. [Q36] |  | 2.98 | 0.003 |  | 0.60 | 1.000 |  | 2.39 | 0.051 |  |
| Clinical practicability / relevance |  |  |  |  |  |  |  |  |  |  |
| The Living Guideline seems to be more informative than the previous print version. [Q37] |  | 3.50 | 0.001 |  | 0.67 | 1.000 |  | 2.85 | 0.013 |  |
| The Living Guideline seems to be more practical than the previous print version. [Q38] |  | 4.12 | <0.001 |  | 0.64 | 1.000 |  | 3.51 | 0.001 |  |
| I can imagine that a Living Guideline would be a valuable tool in my everyday clinical practice. [Q39] |  | 5.16 | <0.001 |  | 3.03 | 0.007 |  | 2.06 | 0.118 |  |
| General attitude |  |  |  |  |  |  |  |  |  |  |
| The advantages of a Living Guideline over a print version are evident to me. [Q40] |  | 4.04 | <0.001 |  | 1.84 | 0.195 |  | 2.17 | 0.091 |  |
| I would prefer a Living Guideline to the previous print version. [Q41] |  | 4.33 | <0.001 |  | 2.16 | 0.093 |  | 2.13 | 0.099 |  |
| Mean – positive attitude Living Guideline* |  |  |  |  |  |  |  |  |  |  |
|  |  | 4.93 | <0.001 |  | 1.66 | 0.291 |  | 3.27 | 0.003 |  |
| *Note.* Agreement was assessed by a 5-point Likert scale (1 = *strongly disagree* to 5 = *strongly agree*). *Z* = standard score. Numbers of questions are displayed in square brackets. The complete questionnaire is shown under point 3. *The variable represents the mean agreement rate of the above displayed items. | | | | | | | | | | |

# **Questionnaire**

| **Q** | **A** |  |
| --- | --- | --- |
|  |  | ***Demographic Information/Experience*** |
|  |  |  |
|  |  |  |
| **1** |  | **How old are you?** |
|  |  | Only an integer value may be entered in this field. |
|  |  | Please enter your answer here: |
|  |  |  |
|  |  |  |
| **2** |  | **Which gender do you have?** |
|  |  | Please select only one of the following answers: |
|  | A1 | o   Female |
|  | A2 | o   Male |
|  | A3 | o   diverse |
|  |  |  |
|  |  |  |
| **3** |  | **In which government district of Bavaria do you work?** |
|  |  | Please select only one of the following answers: |
|  | A1 | o Upper Bavaria |
|  | A2 | o Lower Bavaria |
|  | A3 | o Upper Palatinate |
|  | A4 | o Upper Franconia |
|  | A5 | o Middle Franconia |
|  | A6 | o Lower Franconia |
|  | A7 | o Swabia |
|  |  |  |
|  |  |  |
| **4** |  | **What is your profession?** |
|  |  | If more than one answer is correct, please select your primary profession. |
|  |  | Please select only one of the following answers: |
|  | A1 | o   Psychotherapist |
|  | A2 | o   Psychotherapy trainee |
|  | A3 | o   Psychologist |
|  | A4 | o   Social pedagogue |
|  | A5 | o   Specialist in psychiatry and psychotherapy |
|  | A6 | o   Assistant doctor in psychiatry and psychotherapy |
|  | A7 | o   Specialist in psychosomatic medicine and psychotherapy |
|  | A8 | o   Assistant doctor in psychosomatic medicine and psychotherapy |
|  | A9 | o   Specialist in neurology |
|  | A10 | o   Assistant doctor in neurology |
|  | A11 | o   Specialist in general medicine with qualification for psychosomatic primary care |
|  | A12 | o   Specialist in general medicine without qualification for psychosomatic primary care |
|  | A13 | o   Assistant doctor in general medicine |
|  | A14 | o   (Medical) specialist in another discipline |
|  | A15 | o   Assistant doctor in another discipline |
|  | A16 | o   Specialist nurse for psychiatric care |
|  | A17 | o   Qualified nurse |
|  | A18 | o   Occupational therapist |
|  | A19 | o   Sociotherapist |
|  | A20 | o   Social worker |
|  | A21 | o   Sportstherapist |
|  | A22 | o   Art therapist |
|  | A23 | o   Curative education nurse |
|  | A24 | o   Peer-/Recovery attendant |
|  | A25 | o   Other profession |
|  |  |  |
|  |  |  |
| **5** |  | **How many years of working experience do you have?** |
|  |  | Please select your primarily practiced profession. |
|  |  | Please enter your answer here: |
|  |  |  |
| **6** |  | **In which psychotherapeutic discipline do you train or are you training?** |
|  |  | If more answers are correct, please choose the specialisation you primarily apply. |
|  |  | Please select one of the following answers: |
|  | A1 | o   (Cognitive) behavioral therapy |
|  | A2 | o   Psychoanalytical psychotherapy |
|  | A3 | o   Depth psychology oriented psychotherapy |
|  | A4 | o   Conversational psychotherapy |
|  | A5 | o   Gestalt therapy |
|  | A6 | o   Systemic therapy |
|  | A7 | o   Suggestive and autosuggestive therapy forms |
|  | A8 | o   Body oriented therapies |
|  | A9 | o   Other |
|  | A10 | o   Not applicable |
|  |  |  |
|  |  |  |
| **7** |  | **What is your current workplace?** |
|  |  | Please select all applicable answers: |
|  | A1 | o   Other |
|  | A2 | o   University hospital |
|  | A3 | o   Research |
|  | A4 | o   Public hospital |
|  | A5 | o   Non-profit hospital |
|  | A6 | o   Private hospital |
|  | A7 | o   Practice (public) |
|  | A8 | o   Practice (private) |
|  | A9 | o   Practice (training) |
|  |  |  |
|  |  |  |
| **8** |  | **Does your professional work involve or has it ever involved treating and supporting people with mental illness?** |
|  |  | Please choose one of the following answers: |
|  | A1 | o   Yes |
|  | A2 | o   No |
|  |  |  |
|  |  |  |
| **9** |  | **How would you assess your experience regarding the treatment of people with mental illness?** |
|  |  | Please choose only one of the following answers: |
|  | A1 | o   No experience |
|  | A2 | o   Little experience |
|  | A3 | o   Average experience |
|  | A4 | o   Lots of experience |
|  | A5 | o   Very much experience |
|  |  |  |
|  |  |  |
| **10** |  | **Which patient group do you treat primarily?** |
|  |  | Please choose all applicable answers: |
|  | A1 | o   Patients with main diagnosis ICD-10: F2.xx - Schizophrenia, schizotypal and delusional disorders |
|  | A2 | o   Patients with somatic diagnoses |
|  | A3 | o   Patients with other FX.xx diagnoses |
|  | A4 | o   Patients with main diagnosis ICD-10:F4.xx – neurotic, stress and somatoform disorders |
|  | A5 | o   Patients with main diagnosis ICD-10: F3.xx - Mood [affective] disorders |
|  | A6 | o   Patients with main diagnosis ICD-10: F1.xx - Mental and behavioural disorders due to psychoactive substance use |
|  | A7 | o   Patients with main diagnosis ICD-10: F0.xx - Organic, including symptomatic mental disorders |
|  |  |  |
|  |  |  |
| **11** |  | **Do you treat or have you ever treated people with a schizophrenic disorder?** |
|  |  | Please choose one of the following answers: |
|  | A1 | o   Yes |
|  | A2 | o   No |
|  |  |  |
|  |  |  |
| **12** |  | **How do you assess your experience with regard to the treatment of people with a schizophrenic disorder?** |
|  |  | Please choose only one of the following answers: |
|  | A1 | o   No experience |
|  | A2 | o   Little experience |
|  | A3 | o   Average experience |
|  | A4 | o   Lots of experience |
|  | A5 | o   Very much experience |
|  |  |  |
|  |  |  |
|  |  | ***Attitude towards the guideline for schizophrenia (total)*** |
|  |  |  |
|  |  | In the following you will be asked questions about the guideline for schizophrenia. These all refer to the current evidence- and consensus-based AWMF S3 Guideline for Schizophrenia. For better readability, the term "schizophrenia guideline" is used. |
|  |  |  |
| **13** |  | **How familiar are you with the schizophrenia guideline?** |
|  |  | Please choose only one of the following answers: |
|  | A1 | o   Not at all familiar |
|  | A2 | o   Not familiar |
|  | A3 | o   Neither familiar nor unfamiliar |
|  | A4 | o   Familiar |
|  | A5 | o   Very familiar |
|  |  |  |
|  |  |  |
| **14** |  | **Do you generally agree with the recommendations of the schizophrenia guideline?** |
|  |  | Please choose only one of the following answers: |
|  | A1 | o   Yes |
|  | A2 | o   No |
|  | A3 | o   Undecided – I do have enough information, but I have not decided yet |
|  | A4 | o   Undecided – I need more information in order to make up my mind |
|  |  |  |
|  |  |  |
| **15** |  | **The recommendations of the guideline for schizophrenia are on the whole appropriate and feasible.** |
|  |  | Please choose only one of the following answers. |
|  | A1 | o   Strongly disagree |
|  | A2 | o   Disagree |
|  | A3 | o   Neutral |
|  | A4 | o   Agree |
|  | A5 | o   Strongly agree |
|  | A6 | o   No statement because of no knowledge of the schizophrenia guideline |
|  |  |  |
|  |  |  |
| **16** |  | **How high do you estimate the percentage of your patients (without contraindications) who receive treatment according to the recommendations of the schizophrenia guideline?** |
|  |  | Please select only one of the following answers: |
|  | A1 | o   Not applicable |
|  | A2 | o   Please indicate your answer as percentage (0-100): |
|  |  |  |
|  |  | Now you will be asked questions about specific recommendations of the schizophrenia guideline. The recommendations are described as follows: |
|  |  |  |
|  |  | **Recommendation 1** |
|  |  | *Antipsychotics should be offered as low as possible and as high as necessary (lowest possible dosage) within the by international consensus recommended dosage range. Particularly in first-episode patients a low dose should be chosen as they are more sensitive to side effects and respond better to a lower dose.* |
|  |  |  |
|  |  |  |
| **17** |  | **How familiar are you with the recommendation mentioned above?** |
|  |  | Please choose only one of the following answers: |
|  | A1 | o   Not at all familiar |
|  | A2 | o   Not Familiar |
|  | A3 | o   Neither familiar nor unfamiliar |
|  | A4 | o   Familiar |
|  | A5 | o   Very familiar |
|  |  |  |
|  |  |  |
| **18** |  | **Do you agree with the recommendation mentioned above?** |
|  |  | Please choose only one of the following answers: |
|  | A1 | o   Yes |
|  | A2 | o   No |
|  | A3 | o   Undecided – I do have enough information, but I have not decided yet |
|  | A4 | o   Undecided – I need more information in order to make up my mind |
|  |  |  |
|  |  |  |
| **19** |  | **The recommendation above is appropriate and feasible.** |
|  |  | Please choose only one of the following answers: |
|  | A1 | o   Strongly disagree |
|  | A2 | o   Disagree |
|  | A3 | o   Neutral |
|  | A4 | o   Agree |
|  | A5 | o   Strongly agree |
|  |  |  |
|  |  |  |
| **20** |  | **How high do you estimate the percentage of your patients (without contraindications) who receive treatment according to the recommendation mentioned above?** |
|  |  | Please select only one of the following answers: |
|  | A1 | o   Not applicable |
|  | A2 | o   Please indicate your answer as percentage (0-100): |
|  |  |  |
|  |  |  |
|  |  | Now you will be asked questions about specific recommendations of the schizophrenia guideline. The recommendations are described as follows: |
|  |  |  |
|  |  | **Recommendation 2** |
|  |  | *People with schizophrenia (first-onset and multiple-onset) should be offered treatment with antipsychotics for relapse prevention after evaluating individual risk-benefit.* |
|  |  |  |
|  |  |  |
| **21** |  | **How familiar are you with the recommendation mentioned above?** |
|  |  | Please choose only one of the following answers: |
|  | A1 | o   Not at all familiar |
|  | A2 | o   Not Familiar |
|  | A3 | o   Neither familiar nor unfamiliar |
|  | A4 | o   Familiar |
|  | A5 | o   Very familiar |
|  |  |  |
|  |  |  |
| **22** |  | **Do you agree with the recommendation mentioned above?** |
|  |  | Please choose only one of the following answers: |
|  | A1 | o   Yes |
|  | A2 | o   No |
|  | A3 | o   Undecided – I do have enough information, but I have not decided yet |
|  | A4 | o   Undecided – I need more information in order to make up my mind |
|  |  |  |
|  |  |  |
| **23** |  | **The recommendation above is appropriate and feasible.** |
|  |  | Please choose only one of the following answers: |
|  | A1 | o   Strongly disagree |
|  | A2 | o   Disagree |
|  | A3 | o   Neutral |
|  | A4 | o   Agree |
|  | A5 | o   Strongly agree |
|  |  |  |
|  |  |  |
| **24** |  | **How high do you estimate the percentage of your patients (without contraindications) who receive treatment according to the recommendation mentioned above?** |
|  |  | Please select only one of the following answers: |
|  | A1 | o   Not applicable |
|  | A2 | o   Please indicate your answer as percentage (0-100): |
|  |  |  |
|  |  |  |
|  |  | Now you will be asked questions about specific recommendations of the schizophrenia guideline. The recommendations are described as follows: |
|  |  |  |
|  |  | **Recommendation 3** |
|  |  | *In cases of severe weight gain and the need to continue current antipsychotic medication, after implementation of psychotherapeutic and psychosocial interventions, treatment for weight loss should be offered by trying metformin (first choice) or topiramate (second choice) and by taking into account the risks for additional drug treatment.* |
|  |  |  |
|  |  |  |
| **25** |  | **How familiar are you with the recommendation mentioned above?** |
|  |  | Please choose only one of the following answers: |
|  | A1 | o   Not at all familiar |
|  | A2 | o   Not Familiar |
|  | A3 | o   Neither familiar nor unfamiliar |
|  | A4 | o   Familiar |
|  | A5 | o   Very familiar |
|  |  |  |
|  |  |  |
| **26** |  | **Do you agree with the recommendation mentioned above?** |
|  |  | Please choose only one of the following answers: |
|  | A1 | o   Yes |
|  | A2 | o   No |
|  | A3 | o   Undecided – I do have enough information, but I have not decided yet |
|  | A4 | o   Undecided – I need more information in order to make up my mind |
|  |  |  |
|  |  |  |
| **27** |  | **The recommendation above is appropriate and feasible.** |
|  |  | Please choose only one of the following answers: |
|  | A1 | o   Strongly disagree |
|  | A2 | o   Disagree |
|  | A3 | o   Neutral |
|  | A4 | o   Agree |
|  | A5 | o   Strongly agree |
|  |  |  |
|  |  |  |
| **28** |  | **How high do you estimate the percentage of your patients (without contraindications) who receive treatment according to the recommendation mentioned above?** |
|  |  | Please select only one of the following answers: |
|  | A1 | o   Not applicable |
|  | A2 | o   Please indicate your answer as percentage (0-100): |
|  |  |  |
|  |  |  |
|  |  | Now you will be asked questions about specific recommendations of the schizophrenia guideline. The recommendations are described as follows: |
|  |  |  |
|  |  | **Recommendation 4** |
|  |  | *People with schizophrenia should be offered cognitive behavioral therapy.* |
|  |  |  |
|  |  |  |
| **29** |  | **How familiar are you with the recommendation mentioned above?** |
|  |  | Please choose only one of the following answers: |
|  | A1 | o   Not at all familiar |
|  | A2 | o   Not Familiar |
|  | A3 | o   Neither familiar nor unfamiliar |
|  | A4 | o   Familiar |
|  | A5 | o   Very familiar |
|  |  |  |
|  |  |  |
| **30** |  | **Do you agree with the recommendation mentioned above?** |
|  |  | Please choose only one of the following answers: |
|  | A1 | o   Yes |
|  | A2 | o   No |
|  | A3 | o   Undecided – I do have enough information, but I have not decided yet |
|  | A4 | o   Undecided – I need more information in order to make up my mind |
|  |  |  |
|  |  |  |
| **31** |  | **The recommendation above is appropriate and feasible.** |
|  |  | Please choose only one of the following answers: |
|  | A1 | o   Strongly disagree |
|  | A2 | o   Disagree |
|  | A3 | o   Neutral |
|  | A4 | o   Agree |
|  | A5 | o   Strongly agree |
|  |  |  |
|  |  |  |
| **32** |  | **How high do you estimate the percentage of your patients (without contraindications) who receive treatment according to the recommendation mentioned above?** |
|  |  | Please select only one of the following answers: |
|  | A1 | o   Not applicable |
|  | A2 | o   Please indicate your answer as percentage (0-100): |
|  |  |  |
|  |  |  |
|  |  |  |
|  |  | **Explanation - Living Guideline** |
|  |  |  |
|  |  | Please read the following paragraph about Living Guidelines carefully. The information presented is important for answering the following questions. |
|  |  |  |
|  |  | *A Living Guideline can be understood as an optimized process of guideline development by continuously updating recommendations. In contrast to updating non-living guidelines, with a living guideline individual recommendations are updated rather than the whole guideline. The aim is to facilitate decision making through a timely adaptation of recommendations based on current evidence. Furthermore, a living guideline enables the involvement of users, patients and relatives in the guideline development process at any time.* |
|  |  | *Digital, internet-based systems, such as the "MAGICapp" platform used for the SISYPHOS project make the development of a living guideline possible. Living guidelines are usually updated once a year.* |
|  |  | *In the following, screenshots of the "MAGICapp" are presented to illustrate a Living Guideline. We would like to ask you to look at them carefully, in order to answer questions about the format of Living Guidelines afterwards (e.g. with regard to clarity and comprehensibility).* |
|  |  |  |
|  |  | Screenshot of the MAGICapp as an example of a Living Guideline – in the picture you see the table of contents. We would like to ask you to look at the illustration carefully in order to answer questions about the Living Guideline format afterwards. |
|  |  |  |
|  |  | 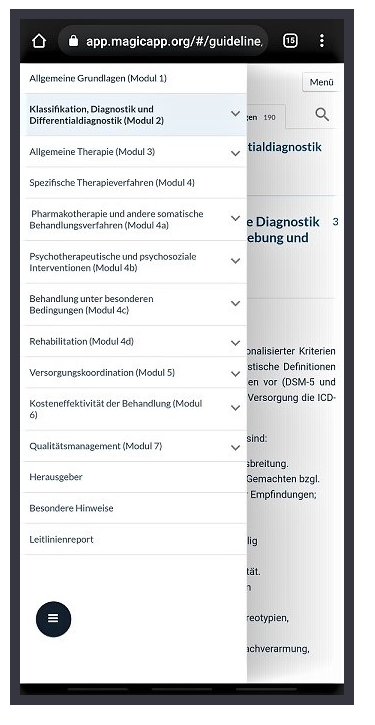 |
|  |  |  |
|  |  | Screenshot of the MAGICapp as an example of a Living Guideline – in the picture you see the guideline recommendation about social skills training and cognitive remediation. |
|  |  | We would like to ask you to look at the illustration carefully in order to answer questions about the Living Guideline format afterwards. |
|  |  | 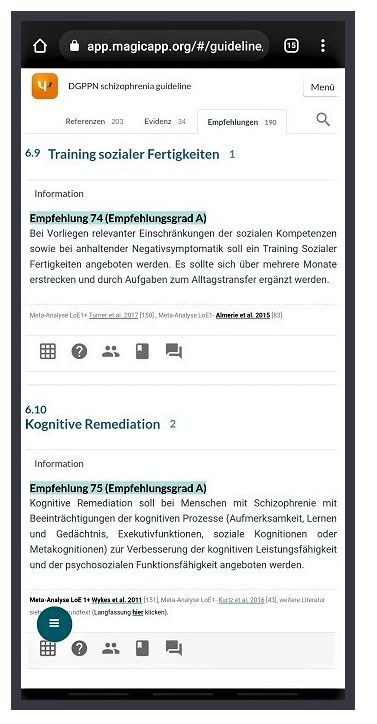 |
|  |  |  |
|  |  |  |
|  |  | Screenshot of the MAGICapp as an example of a Living Guideline – in the picture you see the comparison of two treatment options (treatment as usual vs. social skills training). This graphical depiction can be used to support shared decision making. |
|  |  | We would like to ask you to look at the illustration carefully in order to answer questions about the Living Guideline format afterwards. |
|  |  | 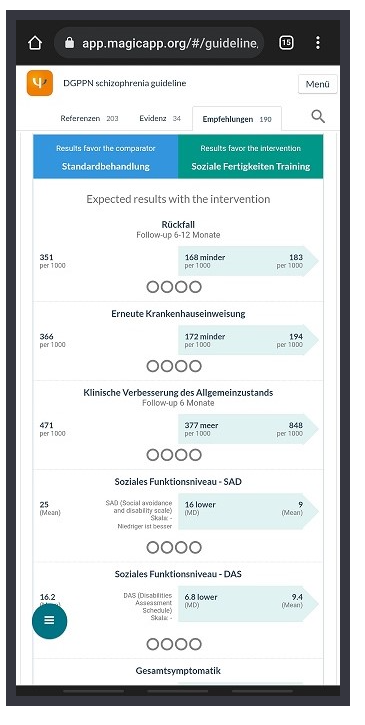 |
|  |  |  |
|  |  |  |
|  |  |  |
|  |  |  |
|  |  | ***Attitude – Living Guideline*** |
|  |  |  |
|  |  |  |
|  |  | In the following we want to investigate the attitude towards Living Guidelines. |
|  |  | In case you do not have any immediate experience with the Living Guideline format, please refer to the impression you received from the shown screenshots. |
|  |  |  |
|  |  | **Please rate the extent to which you agree with the following statements:** |
|  |  | Please choose the correct answer for each item: |
|  | A1 | o   Strongly disagree |
|  | A2 | o   Disagree |
|  | A3 | o   Neutral |
|  | A4 | o   Agree |
|  | A5 | o   Strongly agree |
|  |  |  |
| 33 |  | I find the layout appealing and the content clearly presented. |
| 34 |  | I can imagine getting along well with the Living Guideline. |
| 35 |  | I would need a training in order to be able to use the Living Guideline. |
| 36 |  | The Living Guideline seems clearer to me than the previous print version. |
| 37 |  | The Living Guideline seems to be more informative than the previous print version. |
| 38 |  | The Living Guideline seems to be more practical than the previous print version. |
| 39 |  | I can imagine that a Living Guideline would be a valuable tool in my everyday clinical practice |
| 40 |  | The advantages of a Living Guideline over a print version are evident to me. |
| 41 |  | I would prefer a Living Guideline to the previous print version. |
|  |  |  |
|  |  |  |
|  |  | ***Barriers*** |
|  |  |  |
|  |  | The following questions are referred to individual barriers using the Schizophrenia Guideline - both in terms of the familiar print format and in terms of the Living Guideline format. |
|  |  | Please rate the extent to which you agree with the following statements. In case you do not have any immediate experience with the living guideline format, please refer to the impression you received from the shown screenshots. |
|  |  |  |
|  |  | **Please select the applicable answer for each item - both for the print version and the Living Guideline format:** |
|  | A1 | o   Strongly disagree |
|  | A2 | o   Disagree |
|  | A3 | o   Neutral |
|  | A4 | o   Agree |
|  | A5 | o   Strongly agree |
|  |  |  |
| 42 |  | I have heard of the corresponding guideline format before.* |
| 43 |  | I have experience using the guideline format.* |
| 44 |  | I am confident to use the guideline format or to learn how to use it.* |
| 45 |  | I have sufficient skills to use the guideline format.* |
| 46 |  | There is not enough training/education on how to use guidelines. |
| 47 |  | The use of the format leads or would lead to an increase in the quality of my clinical work.* |
| 48 |  | I lack motivation to deal with the guideline format. |
| 49 |  | The use of the corresponding guideline format leads or would lead to a restriction of my freedom of therapy. |
| 50 |  | The presentation / structure of the corresponding format is / seems confusing. |
| 51 |  | The guideline in the corresponding format is / seems too long and extensive. |
| 52 |  | I know where I can / could find the guideline in the corresponding format.* |
| 53 |  | Working with the corresponding format does not seem to me to be sufficiently proven in clinical setting. |
| 54 |  | Due to lack of time resources (e.g. due to a high workload) the use of the guideline format seems to be difficult. |
| 55 |  | Patients are critical of the guideline format. |
|  |  | **Items were inverted for the related analyses on barriers.* |
|  |  |  |
|  |  | ***Facilitating Factors*** |
|  |  |  |
|  |  | The following questions refer to facilitating factors using the Schizophrenia Guideline - both with regard to the familiar print format and with regard to the Living Guideline format. |
|  |  |  |
|  |  | Please rate the extent to which you agree with the following statements. In case you do not have any immediate experience with the living guideline format, please refer to the impression you received from the shown screenshots. |
|  |  |  |
|  |  |  |
|  |  | **Please select the correct answer for each item - both for the print version and the Living Guideline format:** |
|  | A1 | o Strongly disagree |
|  | A2 | o Disagree |
|  | A3 | o Neutral |
|  | A4 | o Agree |
|  | A5 | o Strongly agree |
|  |  |  |
| 56 |  | I would like to have (more) training/education on working with the guideline format. |
| 57 |  | I would like to see the feedback from the patient’s perspective timely worked in, e.g. regarding the tolerability of medications. |
| 58 |  | I would like to have (more) training for patients and their relatives regarding the use of the guideline. |
| 59 |  | I would like to have the possibility to use the guideline for shared decision making (joint and equal decision making regarding treatment, e.g. with the help of information material, graphics, etc.). |
| 60 |  | I would like to have the practitioners to be more involved in the development of the guideline (e.g. with regard to content or formal design). |
| 61 |  | I would like to have a firm implementation of the guideline in the training / further education. |
| 62 |  | I would like to have clinical conditions more considered (e.g., comorbidities, complex courses) in the content of the guideline. |
| 63 |  | I would like to be supported by quality management. |
| 64 |  | I would like to have a provision of tablets / smartphones for the use of the guidelines in everyday clinical practice. |
| 65 |  | I would like to have simpler and easier understandable language. |
| 66 |  | I would like to have a short and concise version with the essential treatment recommendations (e.g. without the elaboration of underlying evidence). |
| 67 |  | I would like to have short, clear treatment checklists |
| 68 |  | I would like to be notified in case there are changes |
| 69 |  | I would like to have increased awareness of the benefits of the guideline (e.g., in the form of promotional activities). |
| 70 |  | I would like a version tailored to my treatment services or professional group. |
|  |  |  |
|  |  |  |
|  |  | ***Other questions*** |
|  |  |  |
|  |  | Finally, we would like to explore your preferences using the Living Guideline and similar formats. |
|  |  |  |
| **71** |  | **The recommendations of the Living Guideline should ideally be updated at the following intervals:** |
|  |  | Please select only one of the following answers: |
|  | A1 | o Quarterly |
|  | A2 | o Semiannually |
|  | A3 | o Annually |
|  | A4 | o Less frequently than once a year |
|  |  |  |
|  |  |  |
| **72** |  | **The Living Guideline should inform me about new and relevant research results at regular intervals (push notification e.g. to my smartphone, email notification).** |
|  |  | Please select only one of the following answers: |
|  | A1 | o Strongly disagree |
|  | A2 | o Disagree |
|  | A3 | o Neutral |
|  | A4 | o Agree |
|  | A5 | o Strongly agree |
|  |  |  |
|  |  |  |
| **73** |  | **If there were notifications about new and relevant research findings: What would be an appropriate time interval for you?** |
|  |  | Please select only one of the following answers: |
|  | A1 | o Immediately when guideline content is updated |
|  | A2 | o Quarterly |
|  | A3 | o Semiannually |
|  | A4 | o Quarterly |
|  | A5 | o Less frequently than once a year |
|  | A6 | o I do not wish to receive notification |
|  |  |  |
|  |  |  |
| **74** |  | **How would you rate the maximum update period of 12 months for Living Guidelines?** |
|  |  | Please select only one of the following answers: |
|  | A1 | o Much too short |
|  | A2 | o Too short |
|  | A3 | o Appropriate |
|  | A4 | o Too long |
|  | A5 | o Much too long |
|  |  |  |
|  |  |  |
| **75** |  | **An annual update of recommendations or references to new research findings would put pressure on me to constantly adjust my treatment.** |
|  |  | Please choose only one of the following answers: |
|  | A1 | o Strongly disagree |
|  | A2 | o Disagree |
|  | A3 | o Neutral |
|  | A4 | o Agree |
|  | A5 | o Strongly agree |
|  |  |  |
|  |  |  |
| **76** |  | **An annual update of recommendations or reference to new research would be a relieve as I could be sure not to overlook what is "state of the art."** |
|  |  | Please choose only one of the following answers: |
|  | A1 | o Strongly disagree |
|  | A2 | o Disagree |
|  | A3 | o Neutral |
|  | A4 | o Agree |
|  | A5 | o Strongly agree |
|  |  |  |
|  |  |  |
| **77** |  | **I prefer to use other formats / resources than guidelines to learn about evidence-based treatments (e.g. textbooks).** |
|  |  | Please select only one of the following answers: |
|  | A1 | o Strongly disagree |
|  | A2 | o Disagree |
|  | A3 | o Neutral |
|  | A4 | o Agree |
|  | A5 | o Strongly agree |
|  |  |  |
|  |  |  |
| **78** |  | **Which resource do you use most likely to learn about appropriate treatment options?** |
|  |  | Please select only one of the following answers: |
|  | A1 | o Guidelines |
|  | A2 | o Professional literature (e.g., textbooks) |
|  | A3 | o Scientific journals (e.g., Ärzteblatt) |
|  | A4 | o Further education / congresses |
|  | A5 | o Exchange with colleagues |
|  |  |  |
|  |  |  |
| **79** |  | **How often do you use digital tools / apps in your everyday clinical practice (e.g., to look up medication dosages)?** |
|  |  | Please select only one of the following answers: |
|  | A1 | o Never |
|  | A2 | o Seldom |
|  | A3 | o Occasionally |
|  | A4 | o Often |
|  | A5 | o Always |
|  |  |  |
|  |  |  |
| **80** |  | **If your relative/person of trust developed a schizophrenic disorder: Which three interventions would you recommend?** |
|  |  | Please select all applicable answers: |
|  | A1 | o Outreach treatment (e.g., outpatient psychiatric care, sociotherapy). |
|  | A2 | o Treatment in a “Soteria” |
|  | A3 | o Antipsychotic pharmacotherapy |
|  | A4 | o Electroconvulsive therapy |
|  | A5 | o Psychoeducation |
|  | A6 | o Cognitive behavioral therapy |
|  | A7 | o Training-based interventions from cognitive behavioral therapy / metacognitive training |
|  | A8 | o Family interventions and collaboration with family members / persons of trust |
|  | A9 | o Social skills training |
|  | A10 | o Cognitive Remediation |
|  | A11 | o Psychodynamic or psychoanalytic therapy |
|  | A12 | o Conversational psychotherapy and supportive psychotherapy |
|  | A13 | o Occupational therapy |
|  | A14 | o Art therapies |
|  | A15 | o Exercise therapies |
|  | A16 | o Peer-led interventions (exchange between persons affected and experts from own experience) |
|  | A17 | o Vocational rehabilitation |

# **References**

DGPPN. DGPPN, 2019. S3-Leitlinie Schizophrenie (WWW Document). Dtsch. Gesellschaft für Psychiatr. Psychother. Psychosom. und Nervenheilkd. 2019; Available from: https://www.awmf.org/leitlinien/detail/ll/038-009.html
